# Electromagnetic dipole moments of charged baryons with bent crystals at the LHC

**DOI:** 10.1140/epjc/s10052-017-5400-x

**Published:** 2017-12-05

**Authors:** E. Bagli, L. Bandiera, G. Cavoto, V. Guidi, L. Henry, D. Marangotto, F. Martinez Vidal, A. Mazzolari, A. Merli, N. Neri, J. Ruiz Vidal

**Affiliations:** 1 INFN Sezione di Ferrara and Università di Ferrara, Ferrara, Italy; 2 INFN Sezione di Roma and “Sapienza” Università di Roma, Rome, Italy; 3 IFIC, Universitat de València-CSIC, Valencia, Spain; 4 INFN Sezione di Milano and Università di Milano, Milan, Italy; 5 CERN, Geneva, Switzerland

## Abstract

We propose a unique program of measurements of electric and magnetic dipole moments of charm, beauty and strange charged baryons at the LHC, based on the phenomenon of spin precession of channeled particles in bent crystals. Studies of crystal channeling and spin precession of positively- and negatively-charged particles are presented, along with feasibility studies and expected sensitivities for the proposed experiment using a layout based on the LHCb detector.

## Introduction

The magnetic dipole moment (MDM) and the electric dipole moment (EDM) are static properties of particles that determine the spin motion in an external electromagnetic field, as described by the T-BMT equation [1–3]. Several measurements of baryon MDMs contributed to confirm the validity of the quark model [4]. Measurements of the MDM of heavy baryons, i.e. baryons containing charm or beauty quarks, have never been performed due to the difficulties imposed by the short lifetime of these particles of about $10^{-13}{-}10^{-12}{\,\mathrm {s}}$. These measurements would provide important anchor points for QCD calculations, helping to discriminate between different models [5, 6], and would improve the current understanding of the internal structure of hadrons. The possibility to measure the MDM of positively-charged charm baryons at the Large Hadron Collider (LHC) using bent crystals has been proposed in Refs. [7, 8] and recently revisited [9, 10].

The EDM is the only static property of a particle that requires the violation of parity (*P*) and time reversal (*T*) symmetries and thus, relying on $C\!PT$ invariance, the violation of $C\!P$ symmetry. The EDM of a baryon may arise from the structure of quarks and gluons, and any process involving a photon and a flavour–diagonal coupling. In the Standard Model (SM), contributions to the EDM of baryons are highly suppressed but can be largely enhanced in some of its extensions. Hence, the experimental searches for the EDM of fundamental particles provide powerful probes for physics beyond the SM.

Indirect bounds on charm (beauty) quark EDM are set from different experimental measurements and span over several orders of magnitude, i.e. charm (beauty) EDM $\lesssim 4.4\times 10^{-17}{-} 10^{-15}e\,\mathrm {cm}$ [11–15] ($\lesssim 10^{-17} {-} 2\times 10^{-12} e\,\mathrm {cm}$ [13–16]), depending on different models and assumptions. As an example, an indirect bound on the charm quark EDM is derived from the experimental limit on the neutron EDM to be $\lesssim 4.4\times 10^{-17} e\,\mathrm {cm}$ [11], and a charm quark EDM of comparable magnitude is possible in extensions of the SM [12]. For the beauty quark, indirect EDM limits $\lesssim 2\times 10^{-12} e\,\mathrm {cm}$ [13] and $\lesssim 1.22\times 10^{-13} e\,\mathrm {cm}$ [16] are derived, and a relatively large beauty quark EDM is possible in presence of new physics.

Recently, it has been proposed to search for the EDM of positively-charged charm baryons using bent crystals at the LHC  [17]. Similarly to the MDM case, the method relies on baryons produced by the interaction of $7 \,\mathrm {TeV}$ protons, extracted from the LHC beam, on a fixed target. The baryons are subsequently channeled in a bent crystal. The spin precession of short-lived particles is induced by the intense electromagnetic field between the crystal atomic planes. The EDM and the MDM information can be extracted by measuring the spin polarization of the channeled baryons the end of the bent crystal. This technique can be extended to strange and beauty positively-charged baryons.

In this paper we address several key aspects of this unique experimental program. In Sects. 2 and 4, after introducing the channeling of charged particles in bent crystals, we study the deflection and the spin precession of positively- and negatively-charged baryons in a bent crystal using Geant4 simulations. We assess the experimental technique and study the possibility to extend the EDM searches and MDM measurements to negatively-charged baryons. This would allow to perform tests of the $C\!PT$ symmetry by measuring the MDM of particles and antiparticles. In Sect. 3 we prove that the spin evolution equations describing MDM and EDM effects hold for non-harmonic planar channel potential, therefore spin precession effects for both positively- and negatively-charged particles depend uniquely on the crystal curvature. In fact, the same equations also apply for axial-channeled particles, mostly relevant for negatively-charged particles, although its application to MDM and EDM physics will require further investigation. Section 5 focuses on the description of a possible fixed-target setup installed in front of the LHCb detector. The feasibility of the measurements has been evaluated relying on both parametric and Geant4 simulations along with a geometrical model of the detector. Finally, Sect. 6 presents sensitivity studies for EDM searches and MDM measurements of ${{\varLambda } ^+_{c}}$, ${\varXi ^+_{c}}$ charm baryons, ${\overline{\varXi }^+_{b}}$, ${\overline{{\varOmega }}^+_{b}}$ beauty antibaryons, and $\overline{\varXi }^+$, $\overline{{\varOmega }}^+$ strange antibaryons. Baryons and antibaryons will be referred hereafter generically as baryons, unless otherwise stated.

## Channeling of multi-TeV charged particles

In a crystal the strong electric field experienced by a charged particle in the proximity of the ordered structure of the atoms exerts a strong confinement force onto the particle itself. The particle trajectory can be bound to stay parallel to a crystalline plane or to an atomic string, which becomes a preferential pathway in the crystal. This phenomenon is called *channeling* and can occur if the angle between the particle trajectory and a crystal plane (*planar * channeling) or a crystal axis (*axial* channeling) is lower than a Lindhard angle $\theta _L = \sqrt{2U_0/(p\beta c)}$, where $U_0$ is the potential-well depth, *p* the particle momentum and $\beta$ its velocity [18, 19]. This process has been studied in laboratory up to the highest available energy, in particular at the LHC where the planar channeling of 6.5$\,\mathrm {TeV}$ protons has been observed [20].

When the crystal is bent, its planes or atomic strings are bent too. The particle pathways are adiabatically bent following the crystal curvature, resulting in a net deflection of the incoming direction by an angle equal to that of crystal bending: charged particle steering is then possible through channeling in bent crystals. Various applications as circular accelerator halo collimation [21–25] or beam extraction from an accelerator ring for fixed-target experiments [26] have been studied and proposed also for the LHC.

Beam steering of positively-charged particles (positive particles in the following) with channeling has progressed significantly over the last years, featuring silicon crystals with about 80% deflection efficiency at an energy of several hundred GeV [27, 28]. Positive particles in channeling condition are repelled by the atomic electric field and follow trajectories that tend to be far from the lattice sites. Negative particles, on the contrary, are attracted by the same field and can repeatedly oscillate across the nuclei of the crystal. For this reason negative particles are more likely to collide with the nuclei of the crystal lattice and therefore can easily escape from a channeling bound state. The average length that a channeled particle traverses before exiting from the planar or axial potential well is called *dechanneling* length $L_d$, being much shorter for negative than for positive charges.

The first measurements of $L_d$ for ultra-high energy negative particles has been done at CERN using relatively short bent silicon crystal at few hundreds $\,\mathrm {GeV}$. It has been measured to be few $\,\mathrm {mm}$ [29–31] using negative hadron and electron beams, and successfully compared with simulations. Therefore, simulations can be used to extrapolate the efficiency of channeling processes to the multi-TeV energy range and for different crystal curvature radii.

For the ultra-relativistic energy range it was also demonstrated the ability to steer negative particle beams through the axial channeling regime with an efficiency above 90% [32, 33]. Indeed, in the case of axial channeling, there are particles which are above the electric field barrier and are not trapped along a single atomic string. Those particles are anyway deflected due to their stochastic scattering with different atomic strings of the crystal, avoiding the fast dechanneling occurring for negative particles. However, reaching a condition of axial alignment for beam steering is relatively more difficult than for planar channeling since the orientation of the crystal with respect to two (and not only one) rotational axes must be found.

The dependence of the channeling efficiency for positive particles on the particle energy, the crystal length, and bending radius is well known for crystals with the length along the beam comparable to $L_{d}$ [19]. The dechanneling length scales almost proportionally to the particle momentum-velocity $p{\beta }$, i.e. $L_{d} \propto p{\beta }$, and can be calculated as

1 $$\begin{aligned} L_{d} = \frac{256}{9\pi ^2} \frac{p\beta c}{\ln (2m_e c^2 \gamma /I)-1} \frac{a_\mathrm{TF}d_p}{Z_i r_e m_e c^2}, \end{aligned}$$

where $m_e$ and $r_e$ are the mass and the classical radius of the electron, *I* is the mean ionization energy, $d_p$ is the interplanar spacing, $a_\mathrm{TF}$ is the Thomas-Fermi screening radius, and $Z_i$ and $\gamma$ are the charge number and boost of the incident particle.

The channeling efficiency under harmonic approximation scales proportionally to $1 - \eta _{c}$, with $\eta _{c} = R_{c}/R$, where *R* is the crystal bending radius and $R_{c} \propto p{\beta }$ is the critical radius for channeling, i.e. the minimum bending radius for which channeling occurs, and holds,

2 $$\begin{aligned} \epsilon (R) = \epsilon (R=\infty )\left( 1-\eta _{c}\right) . \end{aligned}$$

Since the fraction of particles which remains channeled in a bent crystal has to oscillate near the potential well edge, the probability to leave the channeling state increases for small bending radii. Under harmonic approximation for a bent crystal the dechanneling length is shortened by a factor $(1-\eta _{c})$,

3 $$\begin{aligned} L_{d}(R)=L_{d}(R=\infty )\left( 1-\eta _{c}\right) . \end{aligned}$$

Differently, the energy dependence of $L_{d}$ for negative particles is not theoretically known. Contrarily to the dechanneling of positive particles, the large variation of the transverse energy by negative particles in the interaction with atomic nuclei cannot be treated as a stochastic process, and requires a new formalism to be developed.

To quantify the deflection efficiency of the channeling process in bent crystals, Monte Carlo simulations for both positive and negative particles have been carried out using the Geant4 toolkit [34, 35] in a version allowing for crystalline structures [36]. The channeling process is implemented by including Dynecharm++ [37] and Echarm [38] into the Geant4 channeling package [39]. Such model was validated against experimental data for negative pions [30], protons [40–46], electrons [31, 47–50] and positrons [31] in a range of energies spanning from $855 \,\mathrm {MeV}$ (electrons at MAMI) to $400 \,\mathrm {GeV}$ (protons at CERN). Dynecharm++ allows the tracking of a relativistic charged particle inside a crystalline medium via the numerical integration of the T-BMT classical equations of motion [1–3]. The continuum potential approximation proposed by Lindhard is used [18]. Echarm allows the computation if the electrical characteristics of the crystal is within this approximation.

The Geant4 application was developed on top of the 10.3 version of the toolkit, which allows for crystalline structures [36]. The Geant4 physics lists used were the G4HadronElasticPhysics and the G4HadronPhysicsFTFP_BERT and a custom G4EmStandardPhysics_option4 with single scattering instead of multiple-scattering.

**Fig. 1:**
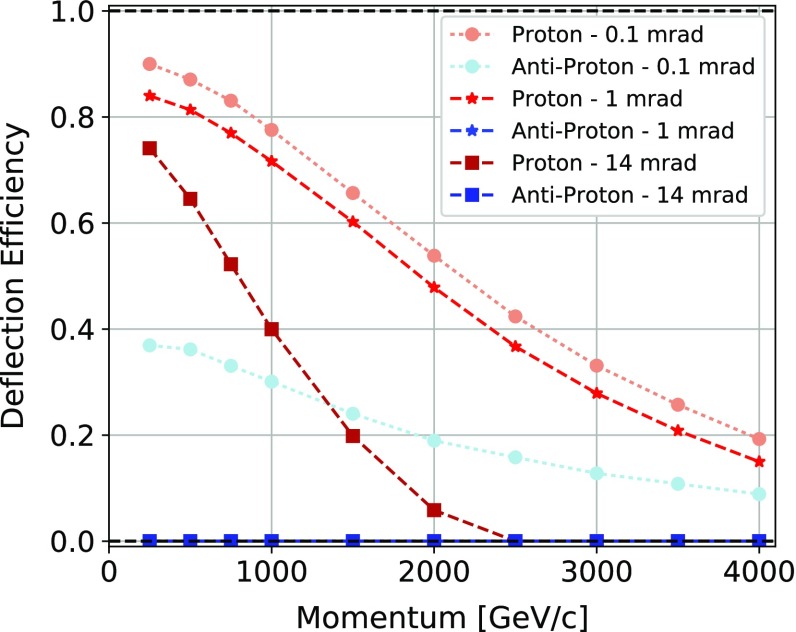
Dependence of the channeling efficiency of protons and antiprotons with the particle momentum for 1 $\,\mathrm {mm}$, 1$\,\mathrm {cm}$ and 7$\,\mathrm {cm}$ long $\mathrm{Si}$ crystals bent along the (110) plane by a 0.1, 1 and 14$\,\mathrm {mrad}$ bending angle, respectively. The curves for the anti-proton interacting with the 1 and 7$\,\mathrm {cm}$ long $\mathrm{Si}$ crystals are superimposed

**Fig. 2:**
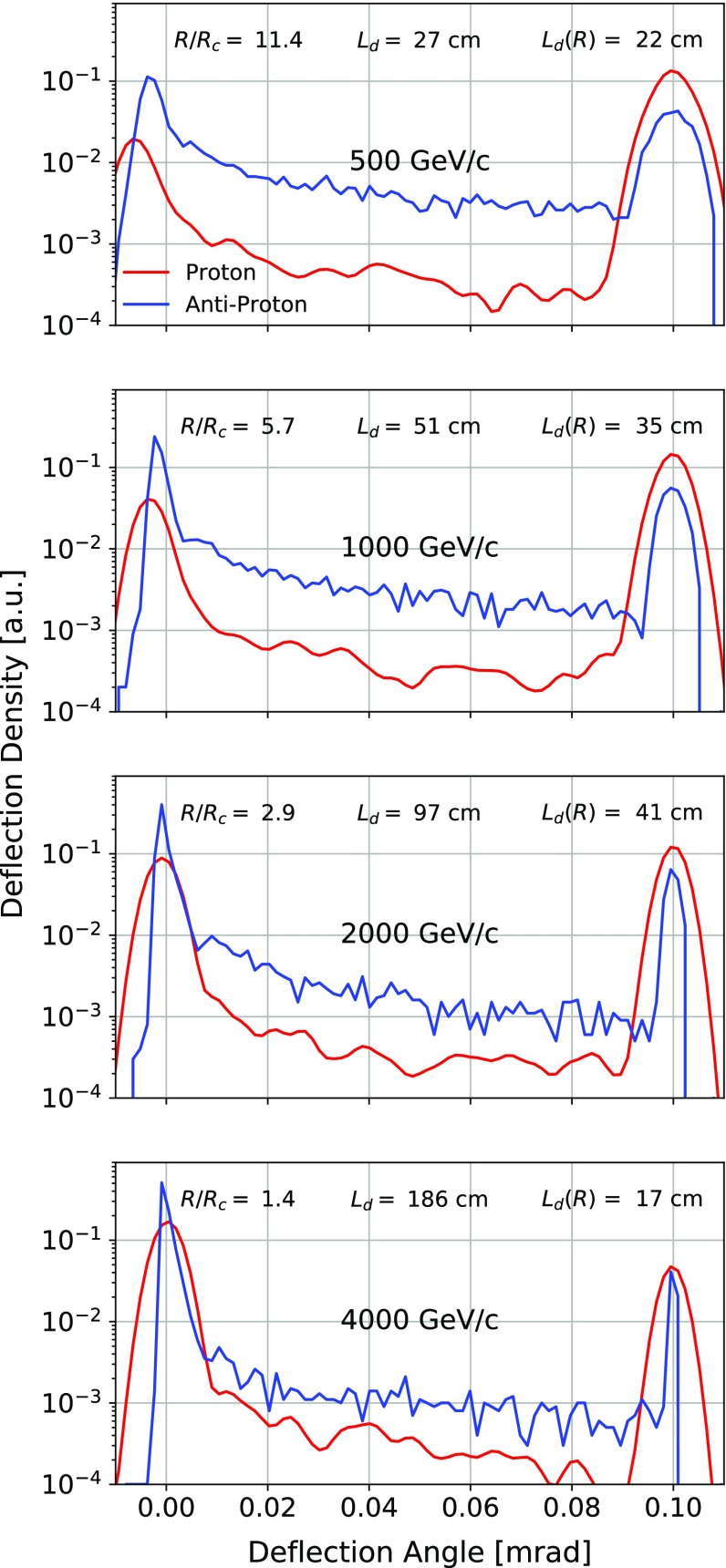
Outgoing angular distributions at various momenta for protons and antiprotons impinging on a 1$\,\mathrm {mm}$ long $\mathrm{Si}$ crystal bent along the (110) plane by a 0.1$\,\mathrm {mrad}$ bending angle. The dechanneling length for positive particles in straight crystals ($L_{d}$) and in the bent crystal ($L_{d}(R)$), along with the critical radius for channeling ($R_{c}$), are calculated for the different momenta following Ref. [19]

Figure 1 shows the dependence of the channeling efficiency for protons and antiprotons with the particle momentum for a 1$\,\mathrm {mm}$, 1$\,\mathrm {cm}$, 7$\,\mathrm {cm}$ long $\mathrm{Si}$ crystal bent along the (110) plane by a 0.1, 1 and 14 $\,\mathrm {mrad}$ bending angle, respectively. The efficiency for positive particles is not spoiled by $L_{d}$, being $L_{d}>13$ cm for all the momenta, but the unfavourable ratio between the critical radius $R_{c}$ and the bending radius is causing it to decrease. Indeed, as illustrated in Fig. 2, this ratio rapidly increases, lowering the deflection efficiency. For negative particles, the deflection efficiency is largely dominated by the crystal length. Therefore, the efficiency remains always lower than for positive particles for all the momenta. Such simulations show that the crystal geometric parameters have to be carefully chosen depending on the energy range in which the crystal has to be operated. In the figure, particles which are not captured under channeling at the crystal entrance are reflected to the opposite side with an angle which depends on the particle momentum [51].

## Spin precession

The spin precession of a charged particle is induced by the interaction of its electromagnetic dipole moments, e.g. MDM and EDM, with external electromagnetic fields. The time evolution of the spin-polarization vector $\mathbf {s}$ is regulated by the T-BMT equation

4 $$\begin{aligned} \frac{d\mathbf {s}}{dt}=\mathbf {s}\times \varvec{\Omega }, \quad \varvec{\Omega }=\varvec{\Omega }_\mathrm{MDM}+\varvec{\Omega }_\mathrm{EDM}+\varvec{\Omega }_\mathrm{TH}, \end{aligned}$$

where the precession angular velocity vector $\varvec{\Omega }$ is composed by three contributions corresponding to the MDM, EDM, and Thomas precession:

5 $$\begin{aligned} \varvec{\Omega }_{\mathrm{MDM}}= & {} \frac{g \mu _B}{\hslash }\left( \mathbf {B} -\frac{\gamma }{\gamma +1}({\varvec{\beta }}\cdot \mathbf {B}){\varvec{\beta }}-{\varvec{\beta }}\times \mathbf {E}\right) ,\nonumber \\ \varvec{\Omega }_{\mathrm{EDM}}= & {} \frac{d \mu _B}{\hslash }\left( \mathbf {E} -\frac{\gamma }{\gamma +1}({\varvec{\beta }}\cdot \mathbf {E}){\varvec{\beta }}-{\varvec{\beta }}\times \mathbf {B}\right) ,\nonumber \\ \varvec{\Omega }_{\mathrm{TH}}= & {} \frac{\gamma ^2}{\gamma +1}\varvec{\beta }\times \frac{d\varvec{\beta }}{dt}= \frac{q}{mc}\bigg [\left( \frac{1}{\gamma }-1\right) \mathbf {B} \nonumber \\&+ \frac{\gamma }{\gamma +1}(\varvec{\beta } \cdot \mathbf {B})\varvec{\beta } - \left( \frac{1}{\gamma +1}-1\right) \varvec{\beta } \times \mathbf {E}\bigg ],\qquad \end{aligned}$$

where $\mathbf {E}$ and $\mathbf {B}$ are the electric and the magnetic fields in the laboratory frame, and *q*, $\gamma$ and $\varvec{\beta }$ are the electric charge, boost and vector velocity of the particle, respectively. The *g* and *d* dimensionless factors, also referred to as the gyromagnetic and gyroelectric ratios, define the magnetic and electric dipole moment of a particle with spin *J* (in Gaussian units) as $\varvec{\mu } = J g \mu _B {\mathbf {s}}$ and $\varvec{ \delta } = J d \mu _B {\mathbf {s}}$, respectively, where $\mu _B=e \hbar / (2 m c)$ is the particle magneton.^1^

**Fig. 3:**
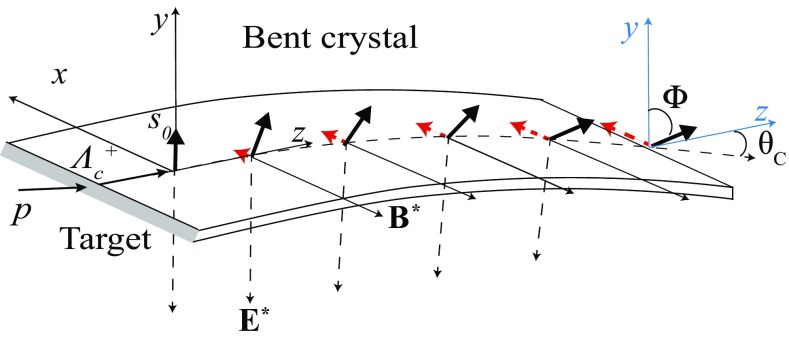
Sketch of the deflection of the ${\varLambda } ^+_{c}$ baryon trajectory and spin precession in a bent crystal. The initial polarization vector ${\mathbf {s}}_{\mathbf {0}}$ is perpendicular to the production plane, along the *y* axis, due to parity conservation in strong interactions. The spin precession in the *yz* and *xy* plane are induced by the MDM and the EDM, respectively. The red (dashed) arrows indicate the (magnified) $s_x$ spin-polarization component proportional to the particle EDM. The $\varPhi$ angle indicates the spin precession due to the MDM

The lifetime of baryons with heavy quark constituents is too short, e.g. the ${\varLambda } ^+_{c}$ baryon lifetime is about $10^{-13}{\,\mathrm {s}}$, for a standard magnet to induce any detectable effect to the spin-polarization vector before they decay. The possibility to measure the MDM of short-lived baryons using channeling in bent crystals was firstly pointed out by V. G. Baryshevsky in 1979. The method is based on the interaction of the MDM of the channeled particles with the intense electric field between crystal atomic planes. As an example, a sketch of the deflection of the ${\varLambda } ^+_{c}$ baryon trajectory and spin precession in a bent crystal is shown in Fig. 3. Charm baryons produced by interaction of protons on a fixed target, e.g. a $\mathrm W$ target, are polarized perpendicularly to the production plane due to parity conservation in strong interactions. The production plane *xz*, also shown in the figure, is determined by the proton and baryon momenta; the latter defines the *z* axis. The initial polarization vector ${\mathbf {s}}_{\mathbf {0}} = (0, s_0, 0)$ is perpendicular to the production plane, along the *y* axis, and at the end of the crystal it is rotated of an angle $\varPhi$. The crystal is bent in the *yz* plane by an angle $\theta _C$. The measurement of the MDM of charm baryons using bent crystals has been widely discussed since the 80’s [52–54]. Lately, the possibility of the measurement at LHC energies has been considered [7–9]. Recently, the search for charm baryon EDM using bent crystals at LHC has been proposed [17].

The spin precession of particles channeled in bent crystals was firstly observed by the E761 Collaboration [55]. Using a 800 ${\,\mathrm {GeV}/c}$ proton beam impinging on a Cu target, $\varSigma ^+$ baryons with 375 ${\,\mathrm {GeV}/c}$ average momentum were produced and channeled in two bent crystals with opposite bending angles. The MDM of the $\varSigma ^+$ baryon was measured and proved the viability of this technique for the measurement of the MDM of short-lived particles.

### Planar channeling

In the case of planar channeling, the intense electric field between the crystal planes, $\mathbf {E}$, which deflects charged particles, transforms into a strong electromagnetic field $\mathbf {E^*} \approx \gamma \mathbf {E}$, $\mathbf {B^*} \approx {-}\gamma \varvec{\beta } \times \mathbf {E}/c$ in the instantaneous rest frame of the particle and induces spin precession. In the limit of large boost, the spin precession induced by the MDM in the *yz* plane is [56]

6 $$\begin{aligned} \varPhi \approx \frac{g-2}{2}\gamma \theta _C . \end{aligned}$$

- In order to obtain $\varPhi \approx 1$ rad are required large crystal bending angles $\theta _C\approx 1{-}10 \,\mathrm {mrad}$, and large Lorentz factors $\gamma \approx 10^2{-}10^3$, which can be uniquely achieved at LHC.

The equations describing the spin precession of planar channeled positive particles in presence of MDM and EDM are derived in Ref. [17]. In the limit of large boost, and assuming small EDM effects compared to the main MDM spin precession, a polarization component orthogonal to the bending plane is induced,

7 $$\begin{aligned} s_{x} \approx s_0 \dfrac{d}{g-2} (\cos {\varPhi }-1). \end{aligned}$$

The MDM driven precession taking place in the bending plane is given by

8 $$\begin{aligned} \begin{aligned} s_y&\approx s_0 \cos \varPhi , \\ s_z&\approx s_0 \sin \varPhi . \end{aligned} \end{aligned}$$

**Fig. 4:**
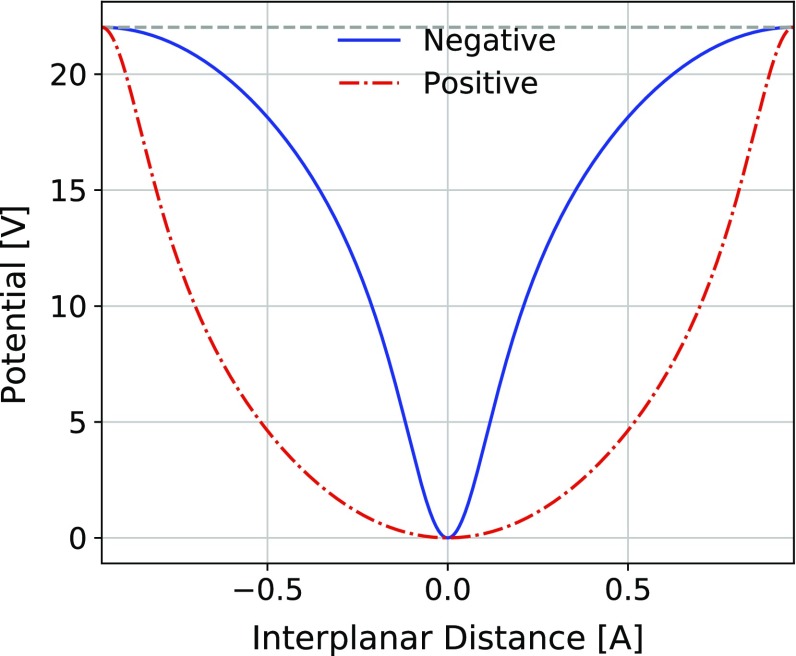
Harmonic (red dash-dotted line) and non-harmonic (blue continuous line) electric potential versus interplanar distance for positive and negative particles in a (110) $\mathrm{Si}$ crystal. The electric potential is extracted from Geant4 simulations. For the sake of comparison the electric potential for negative particles is shifted by half of the interplanar distance

Inside the crystal, positive particles feel a non-zero mean electric field thanks to the centripetal force induced by the crystal bending, and the spin precession depends basically on $\theta _C$. The planar channel potential seen by positive particles can be assumed to be approximately harmonic, as described in Sect. 2. For negative particles this assumption is no more valid since their motion is regulated by a non-harmonic potential, as shown in Fig. 4.

In the following, we demonstrate that in presence of a non-harmonic potential *V*, identical spin precession equations derived for the harmonic potential case hold. We consider the layout of Fig. 3, with the crystal bent along an atomic plane. Polar coordinates are introduced for describing the particle trajectory in the bending plane

9 $$\begin{aligned} y(t) = \rho (t)\cos ({\varOmega } t), \quad z(t) = \rho (t)\sin ({\varOmega } t), \end{aligned}$$

where ${\varOmega }$ is the revolution frequency for the particle traversing the bent crystal, and the electric field described by the planar channel potential $V(\rho )$ is

10 $$\begin{aligned} \mathbf {E} ~=~ \left\{ \begin{aligned} E_x&= 0\\ E_y&= -\frac{dV}{d\rho } \cos ({\varOmega } t)\\ E_z&= -\frac{dV}{d\rho } \sin ({\varOmega } t)~. \end{aligned} \right. \end{aligned}$$

Neglecting EDM contributions, the spin evolution resulting from Eqs. (4), (5), (9) and (10) is

11 $$\begin{aligned} \mathbf {s}(t) ~=~ \left\{ \begin{aligned} s_x(t)&= 0\\ s_y(t)&= s_0\cos \left( \frac{2\mu '{\varOmega }}{\hbar c} \int ^t_0 \rho \frac{dV}{d\rho } dt' \right) \\ s_z(t)&= s_0\sin \left( \frac{2\mu '{\varOmega }}{\hbar c} \int ^t_0 \rho \frac{dV}{d\rho } dt' \right) , \end{aligned} \right. \end{aligned}$$

for the initial condition ${\mathbf {s}_0} = \left( 0, s_0, 0\right)$ and where

12 $$\begin{aligned} \mu '\equiv \frac{g-2}{2}\frac{e\hbar }{2mc}. \end{aligned}$$

The radial coordinate $\rho$ is constant up to $\delta \rho /\rho = \mathcal {O}({\AA }/m) = 10^{-10}$, therefore the spin precession depends on $\int ^t_0 dV/d\rho ~dt'$. Over a complete oscillation in the channel potential the effect of this term is equivalent to that of the electric field in the particle equilibrium radial position $\rho '_0$,

13 $$\begin{aligned} \int ^t_0 \frac{dV}{d\rho } dt'=-E(\rho '_0) t, \end{aligned}$$

which is determined solely by the centripetal force $f_c$ induced by the bent trajectory,

14 $$\begin{aligned} E(\rho '_0) = -\frac{f_c}{e} = -\frac{m\gamma c^2}{e\rho '_0}. \end{aligned}$$

This statement follows by computing

15 $$\begin{aligned} \int ^t_0 \frac{dV}{d\rho } dt' -\left[ -E(\rho _0')t\right] = \int ^t_0 \left( \frac{dV}{d\rho } -\frac{f_c}{e} \right) dt' \end{aligned}$$

for a complete particle oscillation. By changing the integration variable to $d\rho$ and $dt'=d\rho /\dot{\rho }$, then $\dot{\rho }$ is determined by the non-relativistic energy conservation for the radial motion of channeled particles [19]

16 $$\begin{aligned} \frac{1}{2} M\dot{\rho }^2 +e V(\rho ) -f_c\rho = W_{r}, \end{aligned}$$

in which $M=m\gamma$ and $W_{r}$ is the total radial energy, assumed to be constant during a particle oscillation. The relation holds because the longitudinal motion is ultra-relativistic and independent from the radial one, which is non-relativistic since the potential depth is $\mathcal {O}(100 \,\mathrm {eV})\ll m$. The integration boundaries $\rho _{1,2}$ are chosen to be the particle oscillation limits, in which

17 $$\begin{aligned} \frac{1}{2} M\dot{\rho }^2 =0 \leftrightarrow e V(\rho _{1,2}) -f_c\rho _{1,2} = W_{r}~. \end{aligned}$$

Finally, the integral can be trivially computed

18 $$\begin{aligned}&\sqrt{\frac{m}{2e}} \int ^{\rho _2}_{\rho _1} \frac{e\frac{dV}{d\rho } - f_c}{\sqrt{W_{r} +f_c\rho -e V(\rho )}} d\rho \nonumber \\&\quad = -\sqrt{\frac{m}{2e}} \left( \sqrt{W_{r} +f_c\rho _2 -e V(\rho _2)}\right. \nonumber \\&\qquad - \left. \sqrt{W_{r} +f_c\rho _1 -e V(\rho _1)} \right) =0. \end{aligned}$$

Summarizing, spin precession effects given by the actual shape of the planar channel potential cancel out at each particle oscillation and the net spin precession depends uniquely on the crystal curvature. This result generalises the same conclusion previously obtained for harmonic potentials [52, 56]. The spin evolution equations describing MDM and EDM effects, Eqs. (6), (7) and (8), hold as for an harmonic planar channel potential, and in particular for the potential seen by negative particles.

### Axial channeling

The planar channeling efficiency for negative particles is smaller than for positive. It becomes negligibly small for crystals longer than $1\,\mathrm {cm}$ with bending angle larger than $1 \,\mathrm {mrad}$, as shown in Fig. 1. In this case, the spin depolarization effect of particles scattered by crystal axes (planes) provides a possibility for measuring the MDM of negative particles [10]. In case of axial alignment, spin rotation can also be investigated. The phenomenon of axial channeling has been observed for positive and negative particles but it has not been considered for spin precession to date. Here we discuss the possibility to induce spin precession in axial-channeled particles for potential applications in MDM and EDM measurements of charged baryons. We consider the same layout of Fig. 3, in which the crystal is now bent along a crystallographic axis. Polar coordinates are introduced for the bending plane as in Eq. (9) and the electric field described by the axial channel potential $V(x,\rho )$ is

19 $$\begin{aligned} \mathbf {E} ~=~ \left\{ \begin{aligned} E_x&= -\frac{dV}{dx}\\ E_y&= -\frac{dV}{d\rho } \cos ({\varOmega } t)\\ E_z&= -\frac{dV}{d\rho } \sin ({\varOmega } t)~. \end{aligned} \right. \end{aligned}$$

In analogy with planar-channeled particles described in Sect. 3.1, the longitudinal velocity is ultra-relativistic. The velocity components orthogonal to the channel are non-relativistic; their contribution to the spin precession described in Eq. (5), is negligible. The particle velocity inside the bent crystal is therefore simplified as

20 $$\begin{aligned} \beta _x = 0, \quad \beta _y = -\sin ({\varOmega } t), \quad \beta _z = \cos ({\varOmega } t), \end{aligned}$$

in which $\cos ({\varOmega } t)\approx 1$ and $\sin ({\varOmega } t)\approx 0$ to a very good approximation for a crystal bending angle $\theta _C \le 15$ mrad. The spin evolves according to the spin precession equation

21 $$\begin{aligned} \frac{d\mathbf {s}}{dt}=\mathbf {s}\times \varvec{\Omega } = \left\{ \begin{aligned} \frac{ds_x}{dt}&= s_y{\varOmega }_z - s_z{\varOmega }_y\\ \frac{ds_y}{dt}&= s_z{\varOmega }_x - s_x{\varOmega }_z\\ \frac{ds_z}{dt}&= s_x{\varOmega }_y - s_y{\varOmega }_x~, \end{aligned} \right. \end{aligned}$$

with precession vector $\varvec{\Omega }$ following from Eqs. (5), (19) and (20),

22 $$\begin{aligned} \varvec{\Omega } ~=~ \left\{ \begin{aligned} {\varOmega }_x&= -\frac{2\mu '}{\hbar } \frac{dV}{d\rho } - \frac{d\mu _B}{\hbar } \frac{dV}{dx}\\ {\varOmega }_y&= \frac{2\mu '}{\hbar } \frac{dV}{dx} - \frac{d\mu _B}{\hbar } \frac{dV}{d\rho }\\ {\varOmega }_z&= 0~. \end{aligned} \right. \end{aligned}$$

The main difference with respect to the planar channeling case is the presence of the $E_x$ electric field component, which in principle complicates the separation between the MDM and EDM induced spin rotation. Nonetheless, in the following it is shown that the contribution of the *dV* / *dx* terms can be neglected and the spin precession evolution derived for the planar channeling case applies also to axial-channeled particles.

During a particle oscillation the spin can be assumed to be constant since the typical spin precession frequency $\omega =2\mu 'E(\rho '_0)/\hbar \approx 10^{10}$ Hz is three orders of magnitude lower than the oscillation frequency of the particle trapped in the channel, ${\varOmega }_k \approx 10^{13}$ Hz. The two dominant components ${\varOmega }_x \propto dV/d\rho$ and ${\varOmega }_y \propto dV/dx$, describing spin precession in the *yz* and *xz* planes, respectively, can be considered to act independently of each other; namely, the spin rotation in the *yz* plane is not influenced by the spin rotation in the *xz* plane and vice versa. In this case Eq. (13) can be applied to both contributions: while the centripetal force induces a net spin precession in the *yz* plane identical to that of planarly-channeled particles, the effect of *dV* / *dx* mediates to zero over each particle oscillation, since no centripetal force acts in the *x* direction.

The limit of the employed assumption is checked estimating the typical amount of spin precession accumulated during an incomplete particle oscillation, which may lead to an imperfect cancellation of the *dV* / *dx* contribution. This amount is at the order of

23 $$\begin{aligned} \varDelta \approx \frac{2|\mu '|}{\hbar } \int _\mathrm{half} |\mathbf {E}| dt \approx \frac{2|\mu '|}{\hbar } \frac{|\mathbf {E}|}{2{\varOmega }_k} \approx 1.5 \times 10^{-4}, \end{aligned}$$

in which the integration is carried on half of an oscillation. Here, $\mu '$ is taken with $(g-2)/2 = -0.3$ and the ${\varLambda } ^+_{c}$ mass [4]. The typical electric field magnitude of the axial channel $|\mathbf {E}|\approx 4 \times 10^{11}\,\mathrm {eV}/ \,\mathrm {m}$ is estimated as the ratio between the potential depth $\approx 200 \,\mathrm {eV}$ and the channel width $\approx 5 \mathrm {\AA }$ for a Ge crystal, with values taken from Ref. [19]. The oscillation frequency is ${\varOmega }_k = \sqrt{kc^2/eW} \approx 5.4 \times 10^{13}$ for $W = 1\,\mathrm {TeV}$, where the constant describing the potential curvature $k\approx 3.2 \times 10^{22} \,\mathrm {eV}/ \,\mathrm {m} ^2$ for a Ge axial channel is about eight times the value for a $\mathrm{Si}$ axial channel, according to Ref. [19].

Neglecting EDM effects ($d=0$), Eqs. (21) and  (22) show that *dV* / *dx* contributes to the spin component $s_x$ via $ds_x/dt = - s_z{\varOmega }_y$. Since the $s_z$ spin component is not constant during a complete oscillation, the *dV* / *dx* contribution is not exactly zero and can be conservatively estimated in Eq. (24), in which $s_z(t)=\overline{s}_z+\delta s_z(t)$ changes by an amount of order $\varDelta$,

24 $$\begin{aligned} \delta s_x&\approx \frac{2\mu '}{\hbar } \int s_z(t) \frac{dV(t)}{dx} dt \approx \varDelta \frac{2\mu '}{\hbar } \int _\mathrm{half} \frac{dV(t)}{dx} dt \nonumber \\&\approx \varDelta ^2 \approx 2 \times 10^{-8}. \end{aligned}$$

The integrated effect along the whole crystal is conservatively estimated by multiplying $\delta s_x$ by the number of particle oscillations,

25 $$\begin{aligned} \varDelta s_x \approx \delta s_x \frac{L{\varOmega }_k}{c} \approx 3.5 \times 10^{-4}, \end{aligned}$$

in which a crystal length of $L=10\,\mathrm {cm}$ is taken. Such a component does not affect the main MDM spin precession in the *yz* plane and it is negligible compared to the experimental sensitivity on the particle polarization. Indeed, according to the sensitivity studies detailed in Sect. 6, the uncertainty on the $s_x$ spin-polarization component, constituting the EDM signature, will be at the order of $10^{-2}$.

In summary, the spin evolution equations describing MDM and EDM effects, Eqs. (6), (7) and (8), hold also for axial-channeled particles, which is mostly relevant for negative particles with relatively high axial channeling efficiency. Nevertheless, the application of this result to the measurement of the MDM and EDM of particles has to be further studied.

## Geant4 simulations of spin precession

The simulation method based on the numerical integration of the classical equations of motion allows to introduce the modification of the particle spin under the effect of the strong electric field generated by the crystalline lattice. Indeed, the step-by-step variation of the spin is tracked in Geant4 by numeric integration of the T-BMT equation [1–3].

**Fig. 5:**
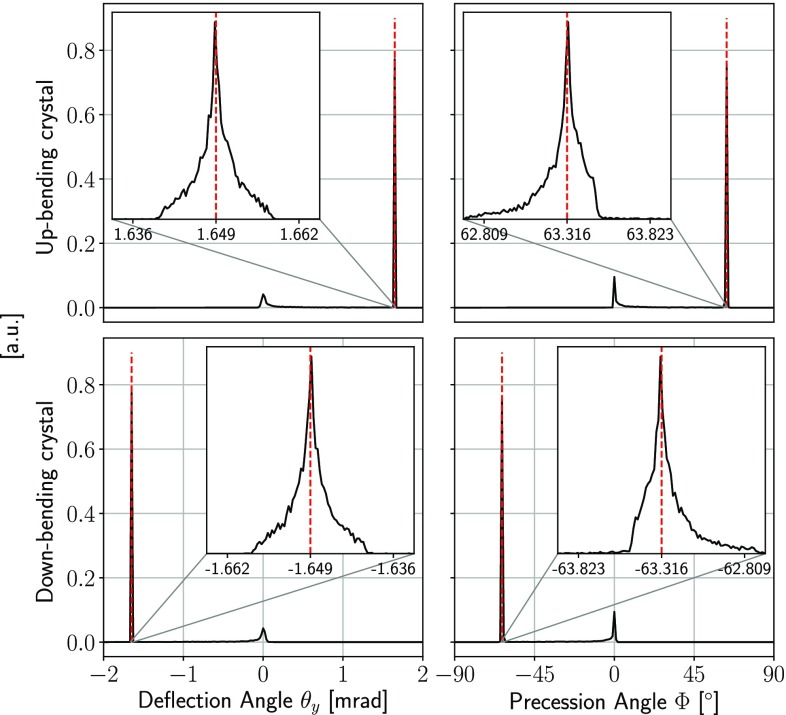
Distributions of the trajectory deflection angle and spin precession angle for $\varSigma ^+$ baryons of 375${\,\mathrm {GeV}/c}$ momentum interacting with 4.5$\,\mathrm {cm}$ long (top) up-bent and (bottom) down-bent crystals, uniformly bent along the (111) plane at $\pm 1.649$ $\,\mathrm {mrad}$ angle. Similar crystals were used for the E761 experiment at FNAL [55]

The Geant4 application for spin precession has been validated against the solely available experimental data provided by the E761 experiment at FNAL [55]. In that experiment, two 4.5$\,\mathrm {cm}$ long $\mathrm{Si}$ crystals bent along the (111) plane were exposed to a $\varSigma ^+$ beam with 375 GeV/c momentum. The deflection angle of the two crystals were $+\,1.649 \,\mathrm {mrad}$ and $-\,1.649 \,\mathrm {mrad}$, with measured precession angles of $-\,72^{\circ } \pm 26^{\circ }$ and $+\,51^{\circ } \pm 23^{\circ }$, respectively. As expected, the spin in the two crystals precesses in opposite directions. The average of experimental values $60^{\circ } \pm 17^{\circ }$ is consistent with the predicted value of $62^{\circ } \pm 2^{\circ }$. A uniformly bent crystal with the E761 geometrical parameters has been implemented within Geant4 and exposed to a monochromatic and perfectly collimated $\varSigma ^+$ beam with 375${\,\mathrm {GeV}/c}$ momentum. Figure 5 shows the distributions of the trajectory deflection angle and of the spin precession angle for both the up- and down-bending crystals. A precession of $+\,63.3^{\circ } \pm 0.2^{\circ }$ and $-\,63.3^{\circ } \pm 0.2^{\circ }$ was obtained for the two cases, respectively, in good agreement with the predicted values of $\pm 63.0^\circ$.

**Table 1:** Average spin precession angle ($\varPhi _\mathrm{sim}$) and EDM polarization component ($s_{x,\mathrm sim}$) obtained from Geant4 simulation compared to the expected values ($\varPhi _\mathrm{exp}$ and $s_{x,\mathrm exp}$, respectively), due to the gyromagnetic factor of the particle expressed as $g'=(g-2)/2$ and the gyroelectric factor $d = 5\times 10^{-2}$, along with the mean channeling deflection efficiency ($\varepsilon _{c}$), for different 1${\,\mathrm {TeV}/c}$ particles impinging on a 1$\,\mathrm {cm}$ long $\mathrm{Si}$ crystal bent along the (110) plane at 1$\,\mathrm {mrad}$ angle. The normalization of the polarization vector ${\mathbf {s}}_{\mathbf {0}}$ has been taken unity, i.e. $s_0=1$

Particle	$g'$	$\varPhi _{\mathrm{exp}}$ [$^{\circ }$]	$\varPhi _{\mathrm{sim}}$ [$^{\circ }$]	$s_{x,\mathrm{exp}}$	$s_{x,\mathrm{sim}}$	$\varepsilon _{c}$ [$\%$]
${\varLambda } ^+_{c}$	$-$0.30	$-$7.518	$\phantom {.}-7.474\pm 0.015$	$\phantom {-}7.17\times 10^{-4}$	$(7.19\pm 0.03)\times 10^{-4}$	$71.0\pm 0.08$
${\overline{\varLambda }} {}^-_{c}$	0.30	7.518	$\phantom {0}\phantom {-}7.59\pm 0.07$	$-\,7.17\times 10^{-4}$	$(-7.20\pm 0.13)\times 10^{-4}$	$0.51\pm 0.07$
$\varXi ^-$	$-$1.92	$-$83.09	$-\,83.0\pm 0.9$	$\phantom {-}1.132\times 10^{-2}$	$\phantom {-}(1.145\pm 0.020)\times 10^{-2}$	$0.47\pm 0.07$
$\overline{\varXi }^+$	1.92	83.09	$\phantom {-}83.21\pm 0.23$	$-\,1.132\times 10^{-2}$	$(-1.149\pm 0.005)\times 10^{-2}$	$70.6\pm 0.08$
${\varOmega } ^-$	$-$2.20	$-$75.38	$-\,75.4\pm 0.6$	$\phantom {-}8.50\times 10^{-3}$	$\phantom {-}(8.51\pm 0.12)\times 10^{-3}$	$0.39\pm 0.6\phantom {0}$
$\overline{{\varOmega }}^+$	2.20	75.38	$\phantom {-}75.53\pm 0.15$	$-\,8.50\times 10^{-3}$	$(-8.51\pm 0.03)\times 10^{-3}$	$70.9\pm 0.8\phantom {0}$
$\varXi ^-_{b}$	$-$1.38	$-$13.65	$-\,13.64\pm 0.14$	$\phantom {-}5.154\times 10^{-4}$	$\phantom {-}(5.15\pm 0.10)\times 10^{-4}$	$0.51 \pm 0.07$
$\overline{\varXi }^+_{b}$	1.38	13.65	$\phantom {-}13.78\pm 0.03$	$-\,5.154\times 10^{-4}$	$(-5.167\pm 0.021)\times 10^{-4}$	$71.0 \pm 0.8\phantom {0}$

**Fig. 6:**
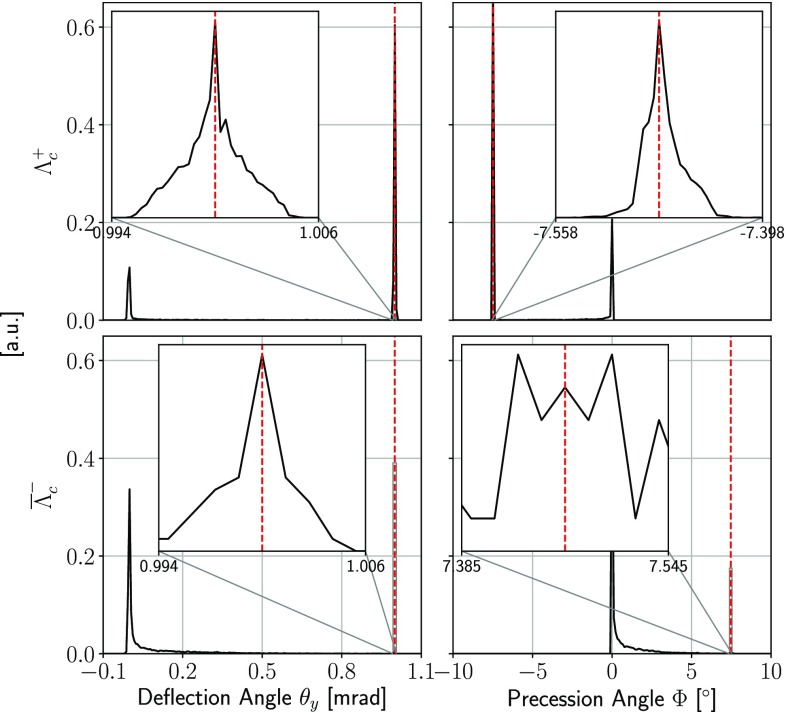
Distributions of the trajectory deflection angle and spin precession angle for (top) ${\varLambda } ^+_{c}$ and (bottom) ${\overline{\varLambda }} {}^-_{c}$ baryons of 1${\,\mathrm {TeV}/c}$ momentum interacting with a 1$\,\mathrm {cm}$ long crystal uniformly bent along the (110) plane at 1$\,\mathrm {mrad}$ angle

The same Geant4 toolkit can also be used to simulate the spin precession of other positive and negative particles in a bent crystal, to compare with the expected analytical values. For this purpose, a 1$\,\mathrm {cm}$ long $\mathrm{Si}$ crystal bent along the (110) plane by a 1$\,\mathrm {mrad}$ bending angle has been used. A beam of short-lived particles with no angular divergence is generated in the simulation immediately before the crystal and the precession angle at the end of the crystal is evaluated. Table 1 shows the simulation results for pairs of short-lived particles and their antiparticles, i.e.  ${{\varLambda } ^+_{c}}/{{\overline{\varLambda }} {}^-_{c}}$, $\varXi ^-/\overline{\varXi }^+$, ${\varOmega } ^-/\overline{{\varOmega }}^+$, and ${\varXi ^-_{b}}/{\overline{\varXi }^+_{b}}$, in presence of MDM and EDM, respectively. Figure 6 shows the distribution of the measured trajectory deflection angles and spin precession angles for the ${\varLambda } ^+_{c}$/${\overline{\varLambda }} {}^-_{c}$ case.

**Fig. 7:**
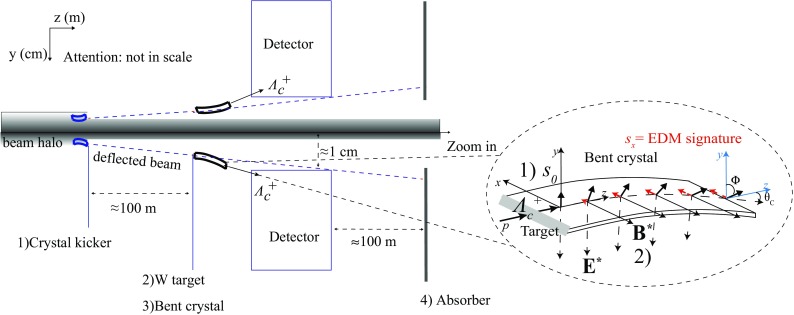
Conceptual layout of the fixed-target setup shown in side view with down- and up-bending crystals. The zoom in shows the spin precession in the down-bending crystal for channeled ${\varLambda } ^+_{c}$ baryons

## The experiment

The MDM and EDM information can be extracted using Eqs. (6), (7) and (8), from the measurement of the spin polarization of channeled baryons at the exit of the crystal, via the study of the angular distribution of final state particles. For $${\varLambda } ^+_{c} $$ decaying to two-body final states such as $$f = {\varDelta } ^{++}{{K} ^-} $$, $$p{{K} ^{*0}} $$, $${\varLambda } (1520){{\pi } ^+} $$ and $${\varLambda } {{\pi } ^+} $$, the angular distribution is described by

26 $$\begin{aligned} \frac{dN}{d{\varOmega }}\propto 1+\alpha _f {\mathbf {s}}\cdot \hat{\mathbf k}, \end{aligned}$$

where $\alpha _f$ is a parity-violating coefficient depending on the final state *f*, $\hat{\mathbf {k}}$ the direction of the final state baryon in the ${\varLambda } ^+_{c}$ helicity frame, ${\varOmega }$ the corresponding solid angle, and $\mathbf{s}$ the ${\varLambda } ^+_{c}$ polarization vector. Equation (26) holds for all spin-1/2 decays into two-body final states with spins $1/2+0$, $1/2+1$ and $3/2+0$.

The initial polarization $s_0$ would require in principle the measurement of the angular distribution for unchanneled baryons. In practice, however, this is not required since the measurement of the three components of the final polarization vector for channeled baryons allows a simultaneous determination of *g*, *d* and $s_0$, up to discrete ambiguities, as discussed in Appendix A. These, in turn, can be resolved exploiting the dependence of the angular distribution with the ${\varLambda } ^+_{c}$ boost $\gamma$.

### Possible experimental layout

The possible experimental layout is based on the double crystal scheme [8] sketched in Fig. 7. It consists of four main elements:

1. two crystal kickers positioned about $100 \,\mathrm {m}$ upstream of the target for up and down deflection at angle $\approx 100{\,\upmu \mathrm {rad}}$ of the 7$\,\mathrm {TeV}$ protons from the LHC beam halo. This technique has been demonstrated to be feasible without affecting the LHC beam lifetime [20];
2. two amorphous $\mathrm W$ targets about $0.5\,\mathrm {cm}$ thick intercepting the deflected proton beam where charm, beauty and strange baryons are produced. The fixed target has to be installed in front of the detector, as close as possible to obtain good vertex resolution;
3. up- and down-bending crystals to induce opposite spin precession to channeled baryons. The use of two crystals with opposite bendings is crucial to prove the robustness of the results and control systematic uncertainties. The $\mathrm W$ target should be attached to the crystal to maximize the yield of channeled baryons;
4. two absorbers positioned downstream of the detector to stop the deflected proton beam and background particles induced by the interactions with the target and crystal materials [57].

To protect against radiation damage and minimise detector occupancies, the design has to guarantee that non-interacting protons and unchanneled particles follow the beam pipe towards the absorbers.

Despite its challenges, the setup is based on two key elements already existing and tested successfully at the LHC: high-purity bent crystals and high-accuracy positioning systems (goniometers). Two bent crystal types with different characteristics are required. The crystal kicker is very similar to that one tested at the LHC  [20], of 4 $\,\mathrm {mm}$ length and $\approx 100{\,\upmu \mathrm {rad}}$ bending angle; the second crystal should have larger angle, order $10 \,\mathrm {mrad}$, as discussed later. The remotely controlled goniometers equipped with the bent crystals are mounted on standard collimation supports and make use of fast plug-in technology, which ensures fast handling of the object. They are based on a piezoelectric actuator and feature angular resolution of $0.1{\,\upmu \mathrm {rad}}$ and linear resolution of $5{\,\upmu \mathrm {m}}$. This is necessary to align the crystal kicker with respect to the beam halo, and position the long-bending crystal to intercept the deflected beam.

Two possible configurations have been considered for the fixed-target and detector setup, referred to hereafter as $\mathsf{S1}$ and $\mathsf{S2}$. The former is based on the upgraded LHCb detector [58], which will become operational in 2021 and will run for the rest of the decade (LHC Run 3 and Run 4), whereas the latter is an hypothetical dedicated detector considered to function at even higher luminosities and providing an angular coverage to minimise the crystal bending angle.

LHCb is a single-arm forward spectrometer [59, 60] dedicated to the study of particles containing $$b $$ or $$c $$ quarks at LHC. The detector tracking system consists of three main devices: a vertex locator (VELO) surrounding the $$p$$ $$p$$ interaction region and a large-area detector (TT) upstream of the dipole magnet inducing an integrated field of about 4 T$$ \,\mathrm {m}$$, and three stations (T1–T3) downstream of the magnet. Particle identification is provided by two ring-imaging Cherenkov detectors, a calorimeter system and muon chambers. With the upgrade, most of the sub-detectors will be replaced [61–63] and a full software based trigger will become operational [64], providing significantly increased efficiencies in hadronic final states and allowing the experiment to operate at higher luminosities. The fixed-target like geometry combined with the capability to reconstruct with good efficiency highly-boosted baryons makes of LHCb the most suitable detector for this proposal.

**Fig. 8:**
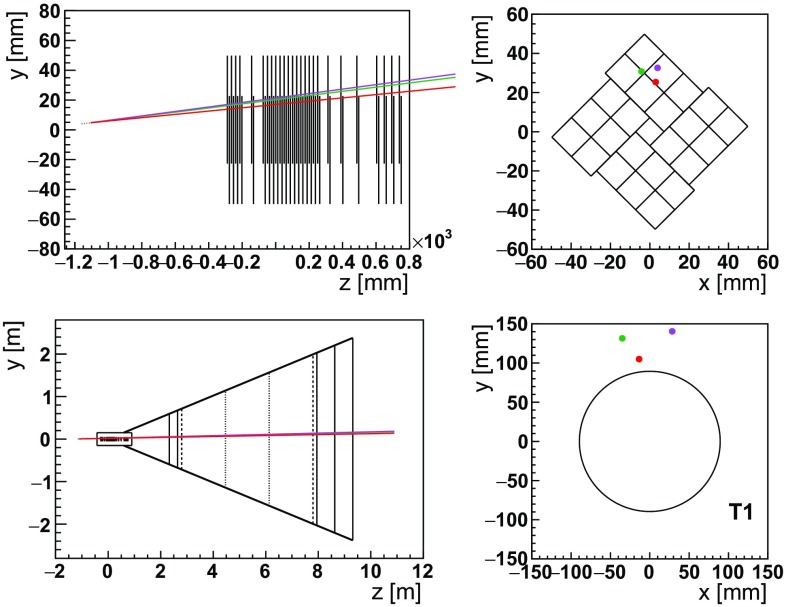
Sketch of a fixed-target ${{\varLambda } ^+_{c}} \rightarrow {p} {{K} ^-} {{\pi } ^+}$ event generated with Pythia at position $(0,0.4,-116)\,\mathrm {cm}$ with a crystal bending $\theta _C=14 \,\mathrm {mrad}$, as seen by the simplified LHCb detector geometry model based on Refs. [61, 62] with a conservative beam clearance of the downstream stations of 30$\,\mathrm {mm}$. The first VELO module is located at $z \approx -29$ $\,\mathrm {cm}$, upstream of the nominal LHC collision point at $z=0$. The points represent the hits of the proton (green), pion (violet) and kaon (red) tracks overlaid in the (top left) side view of the VELO, (top right) front of the last VELO module, (bottom left) schematic side view of the whole LHCb detector, and (bottom right) central area of the T1 station. Events are considered within acceptance when they cross at least three VELO modules and the three T stations. The track bending due to the LHCb dipole magnet is taken into account

To minimise the impact on the interaction region, in the $\mathsf{S1}$ scenario the fixed-target setup is positioned outside of the VELO vessel container [61], $\approx 1.16$ $\,\mathrm {m}$ upstream of the nominal $p$ $p$ collision point and $\approx 0.87$ $\,\mathrm {m}$ upstream of the first VELO module. At this position, a minimal crystal bending angle of about 14$\,\mathrm {mrad}$ is required for channeled baryons to be deflected inside the detector fiducial volume. This follows from a Monte Carlo simulation of fixed-target events using Pythia  [65] together with a simplified geometrical model of the upgraded LHCb detector, as sketched in Fig. 8. For the $\mathsf{S1}$ scenario, a beam intensity of $5\times 10^8{p}/{\,\mathrm {s}}$ impinging the target, and an overall data taking efficiency of 50% is assumed. In this case in six weeks of dedicated detector operations, spanned over several years during the next decade, it would be possible to achieve a statistics of about $10^{15}$ protons on target ($\,\mathrm {PoT}$). Accurate studies of the attainable proton flux are in progress [57], which has to be compliant with the LHC beam lifetime, machine operations and protection. Detector occupancies at such rates are expected to be manageable, as discussed later.

**Fig. 9:**
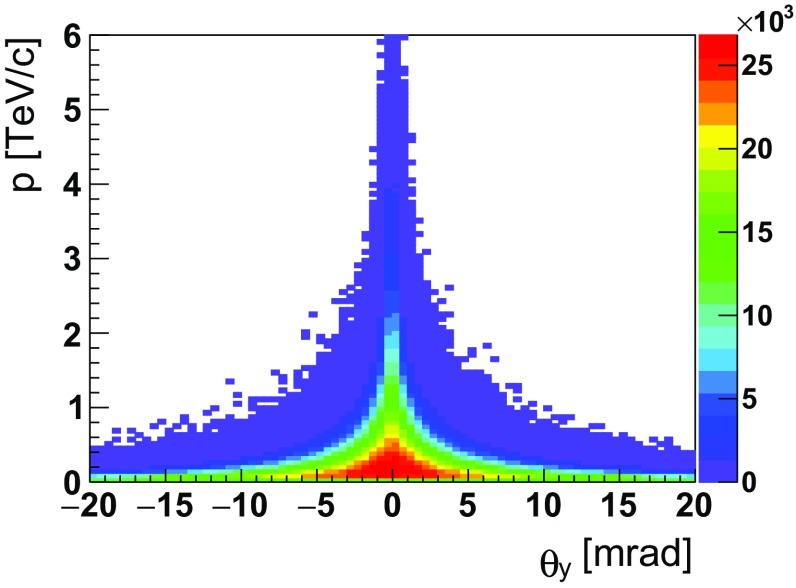
Distribution of polar angle $\theta _y$ versus momentum for ${\varLambda } ^+_{c}$ baryons produced in 7$\,\mathrm {TeV}$ proton beam collisions on protons at rest using Pythia

An increase in proton fluxes, e.g. at High Luminosity LHC (HL-LHC) [66], combined with the design of a dedicated detector capable to afford the higher occupancy levels and longer data taking periods, could potentially offer the opportunity for $\mathsf{S2}$ scenario to integrate $\sim 10^{17}$ $\,\mathrm {PoT}$. Such detector could also extend the angular coverage at larger pseudorapidity to minimise the crystal bending angle, increasing the channeling efficiency, and operate closer to the fixed-target to provide enhanced vertex resolution. Nevertheless, a minimal bending angle of about 5$\,\mathrm {mrad}$ is needed to guarantee a good separation between channeled and unchanneled particles. This value can be inferred from the angular distribution of ${\varLambda } ^+_{c}$ baryons produced by 7$\,\mathrm {TeV}$ protons impinging on the fixed-target, which are highly collimated along the incident proton beam direction, and are isotropically distributed over the azimuthal angle. The polar angle that defines the emission cone is $\propto \gamma ^{-1} \approx 1 \,\mathrm {mrad}$. The corresponding Pythia distribution of polar angle versus momentum, illustrated in Fig. 9, shows that for $|\theta _y|>5 \,\mathrm {mrad}$ and momentum higher than $\approx 1$ $\,\mathrm {TeV}$ there are practically no unchanneled ${\varLambda } ^+_{c}$ baryons.

### Crystal parameters

In Sect. 2 is discussed how the channeling efficiency depends on the crystal parameters and on the momentum range of the particles. In the following, the length *L* and bending angle $\theta _C$ for $\mathrm{Si}$ and $\mathrm{Ge}$ crystals are estimated maximizing the sensitivity of the experiment to the electromagnetic moments, while taking into account detector acceptance. The optimization has been performed using fixed-target ${{\varLambda } ^+_{c}} \rightarrow {p} {{K} ^-} {{\pi } ^+}$ events, produced in 7$\,\mathrm {TeV}$ proton beam collisions on protons at rest using Pythia. The ${\varLambda } ^+_{c}$ channeling has been simulated using a parameterisation based on current theoretical description and channeling measurements, following Ref. [19].

A particle entering the crystal is channeled when its polar angle $\theta _y$ lies within the $(-\theta _L,\theta _L)$ interval, where $\theta _L$ is the Lindhard angle, introduced in Sect. 2. For $p\approx 1$ $\,\mathrm {TeV}$ in Si (Ge) this angle is about 6 (7)${\,\upmu \mathrm {rad}}$, about three orders of magnitude smaller than the angular divergence of ${\varLambda } ^+_{c}$ baryons shown in Fig. 9. Therefore, the trapping efficiency $\varepsilon _{t}$ is largely dominated by the angular opening of the baryons produced in the target. This imposes the crystal to be directly attached to the $\mathrm W$ target. The per-event deflection efficiency is parameterised as

27 $$\begin{aligned} \varepsilon _{c} = (1-\eta _{c})^2 e^{-\theta _C / [ \theta _d \eta _{c} (1-\eta _{c})^2 ]}, \end{aligned}$$

where dechanneling losses inside the bent crystal are described by the factor $(1-\eta _{c})$ introduced in Sect. 2, which accounts for the shortening of the dechanneling length with respect to a straight crystal, and $\theta _d$, which is the ratio of $L_d$ in a straight crystal to $R_c$. The per-event critical radius, $R_{c} = p c/U'(x_c)$, must be below the crystal curvature radius $R=L/\theta _C$, where $U'(x_c)$ is the interplanar electric field at the critical transverse coordinate, below which the particle is lost from channeling mode. For Si 110 (Ge 110), $x_c \approx 0.885~(0.915)$ Å and $U'(x_c) \approx 5.7~(10)$ $\,\mathrm {GeV}$/$\,\mathrm {cm}$  [19].

**Fig. 10:**
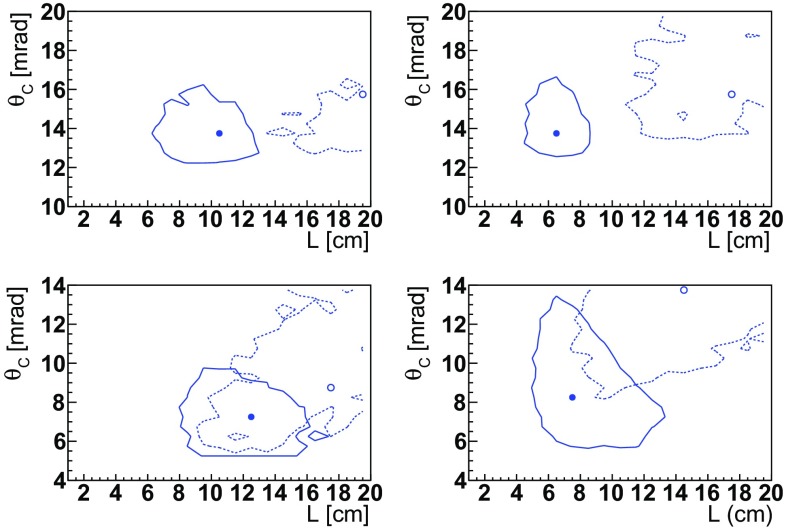
Regions of minimal uncertainty of *d*- and *g*-factors as a function of the crystal parameters *L* and $\theta _C$, for (left) Si and (right) Ge in (top) $\mathsf{S1}$ and (bottom) $\mathsf{S2}$ scenarios. The markers and continuous (dotted) lines represent the minimum uncertainty and regions whose uncertainties on *d* (*g*) are increased by 20% with respect to the minimum, respectively

A scan in the $(L,\theta _C)$ plane is performed to determine the minimal error on the *d* and *g* factors separately. Since there is a wide momentum distribution for the channeled ${\varLambda } ^+_{c}$ baryons, we generate and fit pseudo-experiments using a conditional probability density function constructed with the angular distribution in Eq. (26). Following the discussion at the end of Sec. 5.1, we require the momentum of the ${\varLambda } ^+_{c}$ baryons to be higher than 800 ${\,\mathrm {GeV}/c}$. The dependence of the spin polarization is obtained from Eqs. (7) and (8), assuming $d=0$, $g'=(g-2)/2=-0.3$, $\alpha _f = -0.67$ and $s_0 = -0.6$, as described in more details in Sect. 6. Figure 10 shows regions whose uncertainties on *d* (*g*) are increased by 20% with respect to the minimum, for Si and Ge in both $\mathsf{S1}$ and $\mathsf{S2}$ scenarios. The gyromagnetic ratio prefers higher *L* values, as $\sigma _g \propto 1/\gamma$. These wide regions provide the optimal parameters summarized in Table 2, chosen around the minimum of the gyroelectric factor.

**Table 2:** Bent crystal parameters for $\mathrm{Si}$ and $\mathrm{Ge}$ optimized using charm baryon decays, for the two possible experimental scenarios under consideration. The intervals give approximate regions whose uncertainties on the *d* factor are increased by 20% with respect to the minimum, whereas the central values are chosen for the sensitivity studies discussed in Sect. 6

	S1		S2	
	$\mathrm{Si}$	$\mathrm{Ge}$	$\mathrm{Si}$	$\mathrm{Ge}$
*L* $[\,\mathrm {cm} ]$	[6, 12]	[4, 8]	[8, 15]	[5, 12]
	7	5	12	7
$\theta _C$ $[ \,\mathrm {mrad} ]$	[13, 16]	[13, 16]	[5, 9]	[6, 12]
	14	15	7	8
$R/R_c$	2.85	3.33	9.77	8.75

The parametric approach adopted above to account for dechanneling losses has been validated with Geant4 simulations, discussed in Sects. 2 and 4, using 1$\,\mathrm {TeV}$ ${\varLambda } ^+_{c}$ baryons with no angular divergence. For a 7 (5)$\,\mathrm {cm}$ long $\mathrm{Si}$ ($\mathrm{Ge}$) crystal bent along the (110) plane by a 14 (15)$\,\mathrm {mrad}$ bending angle, the channeling deflection efficiency ${\varepsilon } _c$ is found to be $37.8\pm 0.6$ ($31.7\pm 0.6$)%. In this case the ratio of the optimal crystal bending radius to the critical bending radius, $R/R_c$, is 2.85 (3.33) for the $\mathrm{Si}$ ($\mathrm{Ge}$) crystal. The enhanced channeling efficiency of $\mathrm{Ge}$ compared to $\mathrm{Si}$ is experimentally demonstrated [67–69], and it explains the possibility for $\mathrm{Ge}$ to use shorter crystals with larger bending angles.

The Geant4 toolkit has also been used to evaluate the deflection efficiency in long crystals with large bending angle for negative particles in the$\,\mathrm {TeV}$ energy regime. The efficiency is spoiled by the crystal length, resulting in negligibly small efficiencies even at low momentum, as shown in Fig. 1 for the case of antiprotons traversing a 7$\,\mathrm {cm}$ long $\mathrm{Si}$ crystal with a 14$\,\mathrm {mrad}$ bending. For a 5$\,\mathrm {cm}$ long $\mathrm{Si}$ crystal with 5$\,\mathrm {mrad}$ bending the efficiency amounts to less than 0.1%.

### Detector occupancy

The interaction of the impinging protons on the fixed target might represent a challenge for the detector operations, radiation hardness, and event reconstruction.

The fluence depends mainly on the average number of primary and secondary interactions taking place in the fixed target. The average number of primary interactions can be determined as $$\nu = F p / f$$, where *p* is the probability for a proton to interact in the target material, *F* the rate of impinging protons, and *f* the LHC bunch collision frequency, $$f=11245~\text {Hz}\times 2400=27$$ MHz assuming 2400 bunches. The probability *p* can be estimated itself as $$p = 1-e^{-T/\lambda }$$, where *T* is the thickness of the target material and $$\lambda $$ its nuclear interaction length. For the $$\mathrm W$$ target, $$T=0.5\,\mathrm {cm} $$ and $$\lambda =9.95\,\mathrm {cm} $$ results in $$p=0.05$$, which in turn gives $$\nu = 0.91$$ for a flux $$F=5\times 10^8$$ protons/s. Similarly, for the $$\mathrm{Si}$$ ($$\mathrm{Ge}$$) crystal with $$T=L$$ as given in Table 2 and using $$\lambda = 46.52~(26.86)\,\mathrm {cm} $$ we obtain $$\nu = 2.59~(3.15)$$. Summing together the $$\mathrm W$$ and $$\mathrm{Si}$$ ($$\mathrm{Ge}$$) contributions gives $$\nu = 3.49~(4.05)$$. Similar results are obtained evaluating the number of primary interactions as $$\nu = F N_A \sigma _{{p} {p}} \rho T A_\mathrm{part} / A_T$$, where $$N_A$$ is the Avogadro number, $$\rho $$ the target density, $$A_T$$ the atomic mass, $$\sigma _{{p} {p}}=48$$ $$ \,\mathrm {mb}$$ the total $$p$$ $$p$$ cross section at $$\sqrt{s}\approx 115$$ $$\,\mathrm {TeV}$$, and $$A_\mathrm{part}$$ the number of participant nucleons, estimated using the Glauber model for $${p} $$Pb collisions [70] and rescaling to $$\mathrm W$$ and $$\mathrm{Si}$$ ($$\mathrm{Ge}$$) assuming a spherical geometry for nuclei. These values are about a factor two smaller than for nominal $$p$$ $$p$$ collisions for the LHCb upgrade, $$\nu _{{p} {p}} = \mathcal{L} \sigma _{{p} {p}}/f = 7.6$$, with $$\mathcal{L}=2\times 10^{33}$$ $${\,\mathrm {cm}^{-2}}$$ $${ \,\mathrm {s}^{-1}}$$ and a total $$p$$ $$p$$ cross section at $$\sqrt{s}=14$$ $$\,\mathrm {TeV}$$ of $$\sigma _{{p} {p}}=102.5$$ $$ \,\mathrm {mb}$$.

**Fig. 11:**
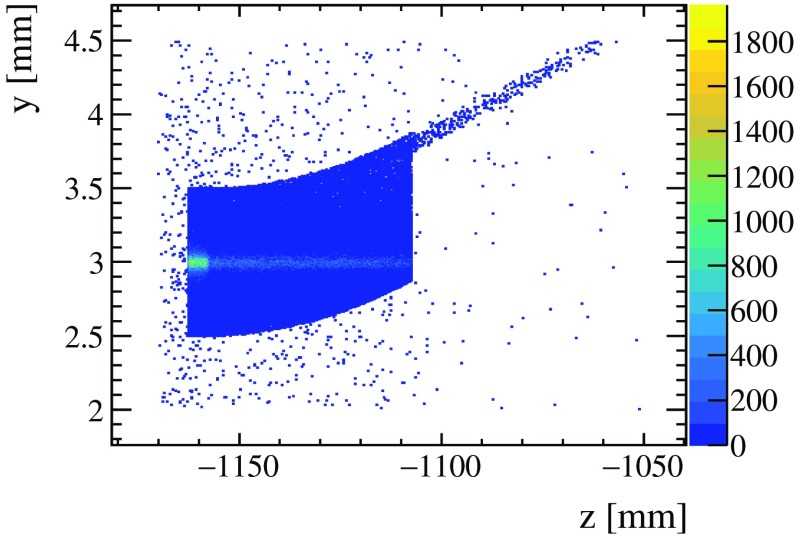
Radiography of the $\mathrm W$ target and $\mathrm{Ge}$ crystal geometries in Geant4, shown in the *zy* view. The distribution represents the origin vertex for stable charged particles from different physics processes (Compton, $\delta$ rays, hadronic interactions and pair production). The presence of the ${\varLambda } ^+_{c}$ decay vertex after the crystal is clearly visible

Secondary interactions have been studied implementing the geometry of the $\mathrm W$ target and $\mathrm{Ge}$ crystal of $\mathsf{S1}$ scenario in the Geant4 framework. Interactions of protons with the $\mathrm W$ target and the $\mathrm{Ge}$ crystal are generated using Epos  [71], tuned to the corresponding average number of primary interactions, whereas ${\varLambda } ^+_{c}$ events use Pythia and EvtGen  [72] to describe their production and decay. Figure 11 illustrates the radiography of the device based on the distribution of stable charged particles. A figure of merit of the occupancy can be obtained from the average number of charged particles within the detector acceptance, using the simplified geometrical model discussed in Sect. 5.1. This value is found to be similar to the corresponding average in the case of nominal $p$ $p$ collisions for the LHCb upgrade.

### Characterization of signal events

**Fig. 12:**
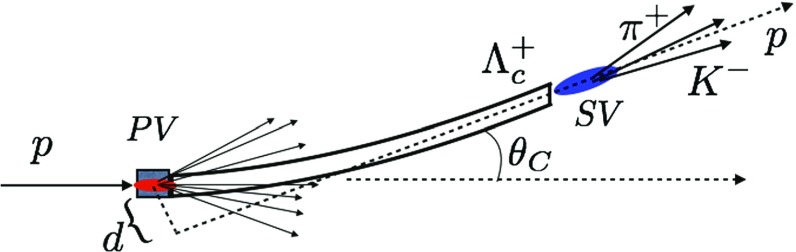
Sketch of a signal event: the ${\varLambda } ^+_{c}$ baryon, produced in the $\mathrm W$ target at the primary vertex ($PV$) of the event, is channeled and deflected at an angle determined by the crystal curvature $\theta _C$. The secondary vertex ($SV$) of the ${\varLambda } ^+_{c}$ baryon decay is outside the crystal, and its position is determined by the vertex of the decay products

A sketch of a ${\varLambda } ^+_{c}$ signal event is shown in Fig. 12. Particles from fixed-target collisions are emitted in a cone with opening angle $\approx 1 \,\mathrm {mrad}$ around the proton direction. Only particles entering the crystal with momenta parallel to the atomic planes within the Lindhard critical angle of few ${\,\upmu \mathrm {rad}}$, $\theta _y \in (-\theta _L,\theta _L)$, are channeled and bent inside the detector acceptance. From the distribution of the polar angle $\theta _y$ versus the momentum, shown in Fig. 9, the fraction of channeled particles is estimated $\approx 10^{-4}$, and represents the hard component of the momentum spectrum. Most of the particles produced in the target are not channeled, remaining confined inside the beam pipe. A small tilt of few Lindhard angles of the crystal atomic planes with respect to the incoming proton direction will avoid channeling of non-interacting protons with no cost in trapping efficiency.

Baryon decay products are also highly collimated, and might be difficult to reconstruct by the detector. For ${{\varLambda } ^+_{c}} \rightarrow {p} {{K} ^-} {{\pi } ^+}$ decays, Pythia simulations indicate that the average angular separation between all pair of tracks range from 1.9 to 2.7$\,\mathrm {mrad}$, depending on their masses. For the $\mathsf{S1}$ scenario, this results in an average radial separation at the first detection layer of about 1.7$\,\mathrm {mm}$, well above the 55${\,\upmu \mathrm {rad}}$ pixel pitch [61]. This would limit the target position a few$\,\mathrm {cm}$ upstream the first sensor in $\mathsf{S2}$ scenario.

The high momentum, the polar angle, and the invariant mass of the outcoming baryons define a distinct signature of signal events. By applying a few selection criteria based on these kinematic variables, e.g. the angle $\theta _y$ (invariant mass) to be within a few $\sigma$ of $\theta _C$ (nominal baryon mass) and the momentum $p \gtrsim 800{\,\mathrm {GeV}/c}$, it would be possible to obtain a high background rejection while retaining most of signal events. For this purpose, the LHCb detector performance are crucial: in particular the estimated track angle resolution of $\approx$25${\,\upmu \mathrm {rad}}$, combined with good momentum and mass resolutions, $\approx 1\%$ [60] and $\approx 20$ ${\,\mathrm {MeV}/c^2}$  [73], respectively. Particle identification is very limited at momentum regime of several hundreds ${\,\mathrm {GeV}/c}$, and is neglected in this study. We assign the particle mass hypothesis based on momentum hierarchy, e.g. for ${{\varLambda } ^+_{c}} \rightarrow {p} {{K} ^-} {{\pi } ^+}$, the highest momentum track is assigned to be the $p$, the second the ${K} ^-$ and the third the ${\pi } ^+$.

With such criteria the vast majority of the signal candidates are produced in the $\mathrm W$ target and decay after the crystal, featuring maximal spin precession. This is because particles produced inside the crystal have lower probability to be channeled, and particles decaying inside the crystal are less bent and have smaller polar angles. The reconstructed invariant mass helps to reject backgrounds from other channeled hadrons having a similar topology, e.g. the more abundant ${{D} ^+} \rightarrow {{K} ^-} {{\pi } ^+} {{\pi } ^+}$ and ${{D} ^+_{s}} \rightarrow {{K} ^+} {{K} ^-} {{\pi } ^+}$ decays for ${{\varLambda } ^+_{c}} \rightarrow {p} {{K} ^-} {{\pi } ^+}$ signal events. Information on the primary and decay vertices could also be exploited to further reject baryons either produced at the beginning or decayed towards the end of the crystal, which might induce a small bias on the spin precession.

Before channeling, a net baryon transverse momentum is needed to define the production plane and have non-zero initial transverse polarization $s_0$, see Fig. 3. This, in turn, requires a non-zero polar angle $\theta _x$, for which there are no restrictions and has a distribution for the hard momentum component similar to that shown in Fig. 9. To prevent initial polarization dilution, the baryon spin rotation has to be determined in bins of $\theta _x$.

**Table 3:** Production cross sections $\sigma$, initial polarizations $s_0$, nuclear modification factors $R_{{p} T}$, and antibaryon-to-baryon ratios ${R_{{\overline{{q}}}/{q}}}$ for $p$ $\mathrm W$ collisions at $\sqrt{s}\approx 115\,\mathrm {GeV}$, along with the anomalous magnetic moment $g'=(g-2)/2$, decay channels, branching ratios $\mathcal {B}$ and decay asymmetry parameters $\alpha _f$, for the different charm, beauty and strange charged baryons. For comparison purposes, the ${\varLambda } ^+_{c}$ case has been considered in the ${\varDelta } ^{++} {{K} ^-}$ and ${\varLambda } {{\pi } ^+}$ final states. Other quantities like particle masses, spins and lifetimes are taken from Ref. [4]. For $\overline{\varXi }^+_{b}$ and $\overline{{\varOmega }}^+_{b}$ antibaryons, $\mathcal {B}$ includes the fragmentation fraction from $\overline{{b}}$ quarks, and $\sigma$ is the total ${p} {p} \rightarrow {b} {\overline{{b}}}$ beauty cross section

Particle	${\varLambda } ^+_{c}$		$\varXi ^+_{c}$	$\overline{\varXi }^+_{b}$	$\overline{{\varOmega }}^+_{b}$	$\overline{\varXi }^+$	$\overline{{\varOmega }}^+$
Decay channel	${\varDelta } ^{++}{{K} ^-}$	${\varLambda } {{\pi } ^+}$	${\varDelta } ^{++}{{K} ^-}$	$\overline{\varXi }^+ {{J/\psi }}$	$\overline{{\varOmega }}^+ {{J/\psi }}$	${\overline{\varLambda }} {{\pi } ^+}$	${\overline{\varLambda }} {{K} ^+}$
				${\overline{\varXi }^0_{c}} {{\pi } ^+}$	${\overline{{\varOmega }}^0_{c}} {{\pi } ^+}$		
Cross section, $\sigma$ [mb]	0.0182		0.0129	$4.67\times 10^{-3}$	$4.67\times 10^{-3}$	3.4	1.03
$|s_0|$	0.6		0.6	0.6	0.6	0.5	0.2
$g'$	$-\,0.3$		$-\,0.3$	1.4	5.8	1.9	2.2
$\mathcal {B}$	$1.09\%$	$0.83\%$	$0.31\%$	$2.9\times 10^{-6}$	$8.3\times 10^{-7}$	$63.83\%$	$43.32\%$
$\alpha _f$	$-\,0.67$	$-\,0.91$	$-\,0.67$	0.91	0.91	0.458	$-\,0.642$
$R_{{p} T}$	$\approx 1$						
${R_{{\overline{{q}}}/{q}}}$	1		1	0.5	0.5	0.8	0.9

## Expected sensitivities

The main contribution to the statistical uncertainty on the *d* and *g* factors of ${\varLambda } ^+_{c}$ baryons, and similarly for all other baryons, can be estimated in the limit of $\gamma \gg 1$ as

28 $$\begin{aligned}&\sigma _d \approx \frac{g-2}{\alpha _f s_0\left( \cos \varPhi -1 \right) }\frac{1}{\sqrt{N_{{\varLambda } ^+_{c}} ^\mathrm{reco}}}~, \nonumber \\&\sigma _{g} \approx \frac{2}{\alpha _f s_0 \gamma \theta _C }\frac{1}{\sqrt{N_{{\varLambda } ^+_{c}} ^\mathrm{reco}}}, \end{aligned}$$

where $N_{{\varLambda } ^+_{c}} ^\mathrm{reco}$ is the number of channeled and reconstructed ${\varLambda } ^+_{c}$ baryons. These estimates assume negligibly small uncertainties on $\theta _C$, $\gamma$ and $s_0$, and follow directly from Eqs. (7) and (8). An alternative approach to assess the sensitivity is to generate and fit pseudo-experiments using a probability density function based on the spin precession motion and angular distribution, Eqs. (7), (8) and (26). The two methods provide consistent results, although the former tends to underestimate the uncertainties by about a factor two compared to the latter.

The number of ${\varLambda } ^+_{c}$ baryons, and similarly for all other baryons, channeled in the bent crystal and reconstructed by the detector can be estimated as

29 $$\begin{aligned} N_{{\varLambda } ^+_{c}} ^\mathrm{reco} = N_{{\varLambda } ^+_{c}} \mathcal {B} ({{\varLambda } ^+_{c}} \rightarrow f)\varepsilon _\mathrm{CH} \varepsilon _{\mathrm{DF}} \varepsilon _{\mathrm{det}}, \end{aligned}$$

where $\mathcal {B} ({{\varLambda } ^+_{c}} \rightarrow f)$ is the branching fraction of the ${\varLambda } ^+_{c}$ decay into the final state *f*, $\varepsilon _\mathrm{CH}$ is the fraction of channeled baryons in the crystal, $\varepsilon _{\mathrm{DF}}$ is a “decay flight” efficiency that accounts for the fraction of channeled ${\varLambda } ^+_{c}$ baryons decaying after the crystal and within the detector fiducial volume, and $\varepsilon _{\mathrm{det}}$ is the detector reconstruction efficiency for ${{\varLambda } ^+_{c}} \rightarrow f$ decays. The number of ${\varLambda } ^+_{c}$ baryons produced with 7$\,\mathrm {TeV}$ protons on a fixed target can be estimated as

30 $$\begin{aligned} N_{{\varLambda } ^+_{c}} = \frac{F t}{S}\sigma ({p} {p} \rightarrow {{\varLambda } ^+_{c}} X) {R_{{\overline{{q}}}/{q}}} N_T , \end{aligned}$$

where *F* is the proton rate, *t* the data taking time, *S* the beam transverse area, $$N_T$$ the number of target nucleons, $$\sigma ({p} {p} \rightarrow {{\varLambda } ^+_{c}} X)$$ the cross-section for $${\varLambda } ^+_{c} $$ production in $$p $$ $$p $$ interactions at $$\sqrt{s}=114.6$$ $$\,\mathrm {GeV}$$ center-of-mass energy, and $${R_{{\overline{{q}}}/{q}}}$$ is the antibaryon-to-baryon ratio for the case of antibaryon production. The number of target nucleons is $$N_T=N_A\rho S T A_N R_{{p} T} /A_T $$, where $$N_A$$ is the Avogadro number, $$\rho $$ (*T*) is the target density (thickness), $$A_T$$ ($$A_N$$) is the atomic mass (mass number), and $$R_{{p} T}$$ is a nuclear modification factor taking into account that the number of participant nucleons of the target nuclei, $$A_\mathrm{part}$$, differs from $$A_N$$ due to nuclear matter effects. For hard processes in the absence of strong final-state interaction $$A_\mathrm{part}$$ scales with $$A_N$$ [70], thus we take $$R_{{p} T} \approx 1$$. For the tungsten target $$\rho =19.25$$ g/cm$$^3$$, $$A_T=183.84$$ g/mol, $$A_N=183.84$$, and $$T=0.5$$ $$\,\mathrm {cm}$$.

**Table 4:** Channeling, survival, decay flight and detector efficiencies, along with the average energy and squared transverse momentum of channeled baryons (before decay flight requirements), for a $\mathrm W$ target with a $\mathrm{Si}$ or $\mathrm{Ge}$ bent crystal in the ${\mathsf{S1}}$ $[{\mathsf{S2}} ]$ scenario. Note that $\varepsilon _{\mathrm{DF}}$ already includes $\varepsilon _{s}$. The sensitivity study based on pseudo-experiments makes use of the complete energy spectrum after channeling, from which $\overline{E}$ and $\overline{p_\perp ^2}$ reported here are obtained. All estimates, except $\varepsilon _{\mathrm{det}}$ (see text), are obtained from samples of charm and strange baryons generated separately for each baryon type from 7$\,\mathrm {TeV}$ proton beam collisions on protons at rest using Pythia. For beauty baryons, we scale the energy of other simulated baryons to obtain an average energy shift estimated assuming a linear dependence with the baryon mass difference

Particle	${\varLambda } ^+_{c}$		$\varXi ^+_{c}$	$\overline{\varXi }^+_{b}$	$\overline{{\varOmega }}^+_{b}$	$\overline{\varXi }^+$	$\overline{{\varOmega }}^+$
Decay channel	${\varDelta } ^{++}{{K} ^-}$	${\varLambda } {{\pi } ^+}$	${\varDelta } ^{++}{{K} ^-}$	$\overline{\varXi }^+ {{J/\psi }}$	$\overline{{\varOmega }}^+ {{J/\psi }}$	${\overline{\varLambda }} {{\pi } ^+}$	${\overline{\varLambda }} {{K} ^+}$
				${\overline{\varXi }^0_{c}} {{\pi } ^+}$	${\overline{{\varOmega }}^0_{c}} {{\pi } ^+}$		
	Si						
$\varepsilon _\mathrm{CH}$ $[\times 10^{-4}]$	1.24 [4.14]		1.04 [3.90]	2.09 [8.91]	2.11 [9.10]	1.75 [5.57]	1.44 [3.84]
$\overline{E}$ $[\,\mathrm {TeV} ]$	1.36 [2.70]		1.24 [2.40]	1.24 [2.44]	1.24 [2.48]	1.12 [1.54]	1.09 [1.33]
$\overline{p_\perp ^2}$ $[{\,\mathrm {GeV}^2/c^2} ]$	1.22 [0.75]		1.09 [1.19]	1.55 [1.25]	1.49 [1.25]	0.20 [0.21]	0.34 [0.32]
$\varepsilon _{s}$ $[\%]$	9.9 [6.9]		31.7 [24.9]	46.3 [41.0]	45.1 [40.1]	99.8 [99.8]	99.5 [99.3]
$\varepsilon _{\mathrm{DF}}$ $[\%]$	9.9 [6.9]	0.42 [0.16]	31.7 [24.7]	46.3 [39.5]	45.0 [38.7]	0.08 [0.05]	0.20 [0.15]
	Ge						
$\varepsilon _\mathrm{CH}$ $[\times 10^{-4}]$	2.32 [5.57]		2.06 [5.18]	3.92 [11.34]	3.98 [11.63]	3.18 [7.34]	2.57 [5.17]
$\overline{E}$ $[\,\mathrm {TeV} ]$	1.37 [2.26]		1.30 [2.07]	1.31 [2.16]	1.32 [2.18]	1.19 [1.51]	1.14 [1.30]
$\overline{p_\perp ^2}$ $[{\,\mathrm {GeV}^2/c^2} ]$	1.16 [1.05]		1.47 [1.09]	1.51 [1.32]	1.52 [1.33]	0.22 [0.22]	0.35 [0.33]
$\varepsilon _{s}$ $[\%]$	20.0 [17.4]		44.9 [40.9]	59.0 [57.4]	57.9 [56.5]	99.9 [99.9]	99.7 [99.6]
$\varepsilon _{\mathrm{DF}}$ $[\%]$	20.0 [17.4]	0.85 [0.52]	44.9 [40.7]	58.9 [56.0]	57.8 [55.2]	0.08 [0.06]	0.20 [0.16]
$\varepsilon _{\mathrm{det}}$ $[\%]$	20	10	20	12	12	10	10

All the necessary inputs and their values as used for the sensitivity study, summarised in Tables 3 and 4, are taken from a combination of measurements, estimates and Monte Carlo simulations, and are discussed in detail in the following, along with the final results.

### Baryon and antibaryon production yields

The $${\varLambda } ^+_{c} $$ and $$\varXi ^+_{c} $$ baryon cross sections can be estimated from the total charm production cross section measured by the PHENIX experiment in $${p} {p} $$ collisions at $$\sqrt{s} = 200\,\mathrm {GeV} $$ [74] rescaled to $$\sqrt{s} = 114.6 \,\mathrm {GeV} $$, assuming a linear dependence on $$\sqrt{s}$$, and the corresponding fragmentation fractions. For the $${\varLambda } ^+_{c} $$ case the fragmentation fraction $$f_{{\varLambda } ^+_{c}} \approx 5.6\%$$ is derived from [74], consistent with theoretical predictions [75]. The $${\varLambda } ^+_{c} $$ baryon branching fractions to $${\varDelta } ^{++}{{K} ^-} $$ and $${\varLambda } {{\pi } ^+} $$ final states are taken from Ref. [4]. The $$\varXi ^+_{c} $$ fragmentation fraction is estimated considering that all known $$c $$-hadron fractions, which amount to about 92%, leave room for the unknown $$\varXi ^+_{c} $$, $$\varXi ^0_{c} $$ and $${\varOmega } ^0_{c} $$ fractions [76, 77]. Assuming $$f_{\varXi ^+_{c}} \approx f_{\varXi ^0_{c}} \gg f_{{\varOmega } ^0_{c}} $$, we obtain $$f_{\varXi ^+_{c}} \approx 4\%$$, which is used to rescale by a factor $$f_{\varXi ^+_{c}}/f_{{\varLambda } ^+_{c}} \approx 0.71$$ the $${\varLambda } ^+_{c} $$ cross section. The absolute $${\varXi ^+_{c}} \rightarrow {\varDelta } ^{++}{{K} ^-} $$ branching fraction is estimated from $$\mathcal {B} ({\varXi ^+_{c}} \rightarrow {p} {{K} ^-} {{\pi } ^+})$$, measured relative to that of $${\varXi ^+_{c}} \rightarrow \varXi ^- {{\pi } ^+} {{\pi } ^+} $$, considering that all known decay modes sum to the total width and assuming that the relative resonant contribution to the $$\varXi ^- {{\pi } ^+} {{\pi } ^+} $$ final state is the same in $$\varXi ^+_{c} $$ and $${\varLambda } ^+_{c} $$ decays. No other quasi-two body $${\varLambda } ^+_{c} $$ or $$\varXi ^+_{c} $$ decays to the final state $${p} {{K} ^-} {{\pi } ^+} $$ are considered for this study. However, there are additional contributions, e.g. $${{\varLambda } ^+_{c}} \rightarrow {{\overline{K}{}} {}^{*0}} {p} $$ and $${{\varLambda } ^+_{c}} \rightarrow \varLambda (1520){{\pi } ^+} $$, with similar branching fractions [4, 78] that can be exploited to improve the sensitivity.

For $\overline{\varXi }^+_{b}$ and $\overline{{\varOmega }}^+_{b}$ baryons produced from 7$\,\mathrm {TeV}$ protons impinging on fixed target, the total beauty cross section can be estimated by rescaling the ${p} {p} \rightarrow {{b} {\overline{{b}}}}$ cross section measured at $\sqrt{s} = 7\,\mathrm {TeV}$ [79]. As a working hypothesis the ratios ${R_{{\overline{{q}}}/{q}}}$ for bottom baryons are assumed to be $\approx 0.5$, on the basis of the results for charm hadron production at lower energies [80–82]. Branching fractions for $\overline{\varXi }^+_{b}$ baryons are known for very few final states. Two suitable two-body decays, requiring a simple two-body angular analysis, are considered. Firstly, the ${\overline{\varXi }^+_{b}} \rightarrow \overline{\varXi }^+ {{J/\psi }}$ decay, where the ${J/\psi }$ and $\overline{\varXi }^+$ can be detected in the dimuon final state and as a positive track, respectively. This decay has been measured and its branching fraction times the $\overline{\varXi }^+_{b}$ fragmentation function is $\approx 6 \times 10^{-7}$ [4]. Secondly, the ${\overline{\varXi }^+_{b}} \rightarrow {\overline{\varXi }^0_{c}} {{\pi } ^+}$ decay, where the charm antibaryon can be reconstructed in the $\overline{\varXi }^+ {{\pi } ^-} {{\pi } ^+} {{\pi } ^-}$, $\overline{\varXi }^+ {{\pi } ^-}$ or ${\overline{p}} {{K} ^+} {{K} ^+} {{\pi } ^-}$ final states. This decay has not been observed but its branching fraction can be estimated by comparing the efficiency corrected signal yields for ${\varXi ^-_{b}} \rightarrow {\varXi ^0_{c}} {{\pi } ^-}$ and ${{\varLambda } ^0_{b}} \rightarrow {{\varLambda } ^+_{c}} {{\pi } ^-}$ decays [83]. The average fragmentation fraction $f_{{\varLambda } ^0_{b}} \approx 7\%$ [84], the measured $\mathcal {B} ({{\varLambda } ^0_{b}} \rightarrow {{\varLambda } ^+_{c}} {{\pi } ^-})$ [4], and the $\varXi ^0_{c}$ branching ratios, estimated similarly to the previous $\varXi ^+_{c}$ case, are used for this calculation. Summing together the contributions of the ${\overline{\varXi }^+_{b}} \rightarrow \overline{\varXi }^+ {{J/\psi }}$ and the ${\overline{\varXi }^+_{b}} \rightarrow {\overline{\varXi }^0_{c}} {{\pi } ^+}$ decays, we obtain a global branching fraction times the fragmentation function as shown in Table 3. Similar decays can be considered for the $\overline{{\varOmega }}^+_{b}$ baryon. In this case, the ${\overline{{\varOmega }}^+_{b}} \rightarrow {\overline{{\varOmega }}^0_{c}} {{\pi } ^+}$ and ${\overline{{\varOmega }}^0_{c}}$ branching ratios are unknown, and we assume the latter to be the same as for $\varXi ^0_{c}$ decays and scale $f_{\overline{{\varOmega }}^+_{b}} \mathcal {B} ({\overline{{\varOmega }}^+_{b}} \rightarrow {\overline{{\varOmega }}^0_{c}} {{\pi } ^+})$ by $\approx 0.29$, from the ratio between $f_{{\varOmega } ^-_{b}} \mathcal {B} ({{\varOmega } ^-_{b}} \rightarrow {\varOmega } ^- {{J/\psi }})$ and $f_{\varXi ^-_{b}} \mathcal {B} ({\varXi ^-_{b}} \rightarrow \varXi ^- {{J/\psi }})$ [4].

For the $$\overline{\varXi }^+$$ and $$\overline{{\varOmega }}^+$$ antibaryons, which contain two and three $$s $$ valence antiquarks respectively, the cross sections are estimated by scaling the $$\varLambda $$ production cross section using the universal strangeness suppression factor at high energies, $$\lambda _s \approx 0.32$$ [85]. In turn, the $$\varLambda $$ production cross section is estimated from the inclusive $${p} {p} \rightarrow {\varLambda } X$$ cross section measured at beam momenta of 158$$\,\mathrm {GeV}$$  [86] and 405$$\,\mathrm {GeV}$$  [87] ($$\sqrt{s}\approx 17.2$$ and 27.6$$\,\mathrm {GeV}$$, respectively). The ratios $${R_{{\overline{{q}}}/{q}}}$$ are taken to be 0.8 and 0.9 for $$\overline{\varXi }^+$$/$$\varXi ^-$$, $$\overline{{\varOmega }}^+$$/$${\varOmega } ^-$$, respectively, as inferred from Au+Au collisions at $$\sqrt{s_{NN}}=130$$ $$\,\mathrm {GeV}$$  [88]. All branching ratios are in this case known [4].

### Efficiencies

The channeling efficiency $\varepsilon _\mathrm{CH}$ in $\mathrm{Si}$ and $\mathrm{Ge}$ crystals includes both the trapping efficiency $\varepsilon _{t}$ and deflection efficiency $\varepsilon _{c}$, and has been estimated separately for each baryon type following the procedure described in Sect. 5.2. The trapping efficiency itself accounts for the angular and momentum divergence of the baryons produced in the target, and is evaluated from the fraction of baryons within the Lindhard angle and momentun $\gtrsim 800{\,\mathrm {GeV}/c}$. Crystal parameters, optimized for charm baryons, are taken to be common for all baryon species.

The decay flight efficiency $\varepsilon _{\mathrm{DF}}$ has two contributions: the survival efficiency, $\varepsilon _{s}$, which accounts for the fraction of channeled baryons decaying after the crystal, and the probability $\varepsilon _{l}$ for long-lived baryons to decay within the VELO region, $\approx 80$ $\,\mathrm {cm}$ downstream of the nominal $p$ $p$ collision point. When one of the baryon decay products is a long-lived $\varLambda$, $\varepsilon _{l}$ also accommodates the probability of the $\varLambda$ to decay before the large-area tracking system upstream the magnet, $\approx 2$ $\,\mathrm {m}$ downstream of the collision point, assuming it takes on average half of the initial baryon momentum. For simplicity, the same requirements are applied for both $\mathsf{S1}$ and $\mathsf{S2}$ scenarios.

The detector efficiency $\varepsilon _{\mathrm{det}}$ can be estimated from the product of the geometrical, trigger and tracking efficiencies, the latter including combinatorics and selection efficiencies. The software-based trigger for the LHCb upgrade detector [64], our $\mathsf{S1}$ scenario, is expected to have efficiency for charm hadrons comparable to the current high level trigger [60], $\approx 80\%$, and similarly for other baryons. A specific trigger scheme for the fixed-target experiment based on the distinct signature of the signal events can enhance the trigger efficiency to $\approx 100\%$. The tracking efficiency is estimated to be 70% per track. Following the discussion in Sect. 5, the geometrical efficiency is taken $\approx 50\%$. For decays to final states including $\varLambda$ baryons we further apply a penalty factor 1 / 2 to account for the additional inefficiencies to reconstruct highly displaced vertices. Note that the inefficiency due to the long lifetime of the $\varLambda$ baryon is separately taken into account in $\varepsilon _{\mathrm{DF}}$, as discussed before.

### Spin polarization of baryons

The asymmetry parameter of the ${{\varLambda } ^+_{c}} \rightarrow {\varLambda } {{\pi } ^+}$ decay has been measured to be $-0.91\pm 0.15$ [4]. For other ${\varLambda } ^+_{c}$ decays no measurements are available but an effective $\alpha _f$ parameter can be calculated from a Dalitz plot analysis of ${{\varLambda } ^+_{c}} \rightarrow {p} {{K} ^-} {{\pi } ^+}$ decays [78], e.g. $\alpha _{{{\varLambda } ^+_{c}} \rightarrow {\varDelta } ^{++}{{K} ^-}} = -0.67\pm 0.30$ [17]. Eventually, a Dalitz plot analysis of ${{\varLambda } ^+_{c}} \rightarrow {p} {{K} ^-} {{\pi } ^+}$ decays would provide the ultimate sensitivity to dipole moments. For the ${\varXi ^+_{c}} \rightarrow {\varDelta } ^{++}{{K} ^-}$ decay the asymmetry parameter is taken to be similar to the ${\varLambda } ^+_{c}$ decay to the same final state, whereas for all beauty antibaryon decays it is assumed to be about the same as for the ${{\varLambda } ^+_{c}} \rightarrow {\varLambda } {{\pi } ^+}$ decay. For the $\overline{\varXi }^+ \rightarrow {\overline{\varLambda }} {{\pi } ^+}$ decay the asymmetry parameter is taken from [4]. The $\overline{{\varOmega }}^+ \rightarrow {\overline{\varLambda }} {{\pi } ^+}$ decay is predominantly parity-conserving and thus has a negligibly small asymmetry parameter [89]. The polarization can be determined in this case from the angular distribution of the antiproton from the $\overline{\varLambda }$ decay, as the $\overline{{\varOmega }}^+$ polarization can be related to the polarization of its $\overline{\varLambda }$ child baryon such that ${\mathbf {s}}_{\overline{{\varOmega }}^+} = {\mathbf {s}}_{{\overline{\varLambda }}}$ [90, 91].

**Fig. 13:**
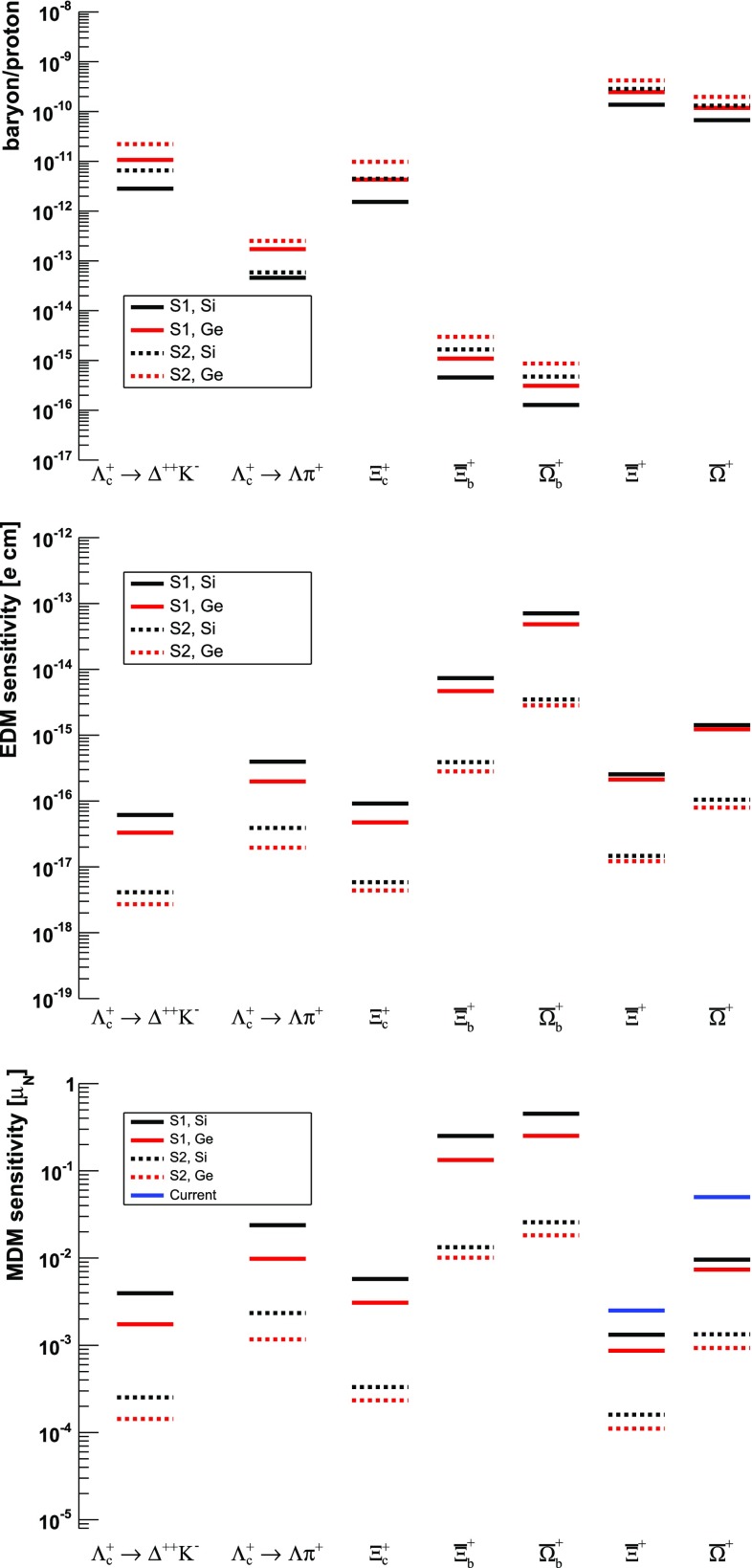
(Top) Estimated yields of channeled and reconstructed signal baryons per incident proton on target, and (middle) EDM and (bottom) MDM sensitivities, for $\mathrm{Si}$ and $\mathrm{Ge}$ with crystal parameters optimized for $\mathsf{S1}$ and $\mathsf{S2}$ scenarios. A total of $10^{15}$ and $10^{17}$ protons on target have been considered for $\mathsf{S1}$ and $\mathsf{S2}$ respectively. For comparison purposes, the ${\varLambda } ^+_{c}$ case has been studied in the ${\varDelta } ^{++} {{K} ^-}$ and ${\varLambda } {{\pi } ^+}$ final states. Blue lines show the sensitivity of the current $\varXi ^-$ and ${\varOmega } ^-$ MDM measurements

The initial polarization of ${\varLambda } ^+_{c}$ particles produced from the interaction of 7$\,\mathrm {TeV}$ protons on a fixed target is unknown. However, a measurement from interaction of 230 GeV/*c* $\pi ^-$ on copper target yields $s_{0} = -0.65^{+0.22}_{-0.18}$ for $\varLambda _c^+$ transverse momentum larger than 1.1 GeV/*c* [92]. Moreover, from data produced in the interactions of 500 ${\,\mathrm {GeV}/c}$ ${{\pi } ^-}$ on five thin target foils (one platinum, four diamond) [78], the polarization of the ${\varLambda } ^+_{c}$ is measured as a function of the ${\varLambda } ^+_{c}$ transverse momentum. The average polarization is about $-0.1$, reaching $-0.7$ for $p_\perp ^2$ between about 1.24 and 5.2$\,\mathrm {GeV} ^2/c^2$. Considering these measurements and the average transverse momentum of channeled ${\varLambda } ^+_{c}$ baryons given in Table 4, we assume $|s_0|=0.6$ for both ${\varLambda } ^+_{c}$ and $\varXi ^+_{c}$ baryons [54]. The same polarization is assumed for the $\overline{\varXi }^+_{b}$ and $\overline{{\varOmega }}^+_{b}$ antibaryons. Similarly, the initial polarization of $\overline{\varXi }^+$ and $\overline{{\varOmega }}^+$ antibaryons produced from the interaction of 7$\,\mathrm {TeV}$ protons on fixed target is unknown. From proton production below the$\,\mathrm {TeV}$ region [90, 91, 93–96], the $\overline{\varXi }^+$ are found to be polarized with the same sign and magnitude as the $\varXi ^-$, increasing about linearly with momentum and reaching $\approx -0.2$ at 250${\,\mathrm {GeV}/c}$, whereas the ${\varOmega } ^-$ is consistent with no polarization. As a working hypothesis initial polarizations of $|s_0|=0.5$ and 0.2 are assumed for $\overline{\varXi }^+$ and $\overline{{\varOmega }}^+$, respectively, considering the large momentum of channeled antibaryons $\approx 1\,\mathrm {TeV}$/c.

Theoretical predictions of $g-2$ for the ${\varLambda } ^+_{c}$ and $\varXi ^+_{c}$ baryons range between $-0.64$ and 0.22 [5, 54], thus a central value $g'=(g-2)/2 =-0.3$ is considered. For $\overline{\varXi }^+_{b}$ and $\overline{{\varOmega }}^+_{b}$ antibaryons we take effective quark mass MDM calculations [6]. For all strange baryons under consideration there exist measurements [4].

### Results

Combining all parameters, measurements and estimates discussed above and summarized in Tables 3 and 4, we obtain the signal yields, normalized to the incident proton flux *F*, shown in (top) Fig. 13. These rates procure the expected EDM and MDM sensitivities reported in (middle and bottom) Fig. 13, for both $\mathrm{Si}$ and $\mathrm{Ge}$ crystals and the two considered experimental scenarios, $\mathsf{S1}$ with $10^{15}\,\mathrm {PoT}$ and $\mathsf{S2}$ with $10^{17}\,\mathrm {PoT}$. Germanium crystals provide in all cases significantly better EDM (MDM) sensitivities, which are for $\mathsf{S1}$ scenario of order $10^{-17}$, $10^{-14}$ and $10^{-16}$ $e\,\mathrm {cm}$ ($10^{-3}$, $10^{-1}$ and $10^{-3}$ $\mu _N$) for charm, beauty and strange baryons, respectively. Here $\mu _N=e\hslash /2m_pc$ is the nuclear magneton, and $m_p$ the proton mass. Sensivities for $\mathsf{S2}$ scenario would improve by about one order of magnitude.

## Conclusions

Electric and magnetic dipole moments of short-lived baryons are powerful probes for physics within and beyond the SM. However, EDM and MDM for charm and beauty baryons have not been accessible to date. A unique opportunity to measure at LHC the EDM and MDM of charm, beauty and strange charged baryons has been discussed here. The experimental setup is based on a fixed-target to be installed in the LHC where protons from the beam halo are deflected using a bent crystal, producing transversally polarized baryons from their interactions with the target. A second bent crystal is positioned after the target where charged baryons that are channeled deflect their trajectory and enter the detector acceptance while rotating their spin. The MDM and EDM information can be inferred from the measurement of the spin-polarization vector after the crystal by analysing the angular distribution of the baryon decay products.

The planar channeling efficiency for multi-$\,\mathrm {TeV}$ particles and the spin precession in bent crystals is studied using Geant4 simulations. The main result is that both positive and negative particles feature spin precession and the results agree with predictions based on analytical calculations, also discussed in Ref. [17]. However, planar channeling efficiency for negative particles is consistently lower than for positive particles, thus much higher statistics is required to perform useful measurements. In that case $C\!PT$ tests based on the MDM for baryons and antibaryons could be performed. The possibility of exploiting axial channeling of negative particles has been briefly discussed but more studies are needed, including Monte Carlo Geant4 simulations, before drawing any conclusion on the possibility to measure electromagnetic dipole moments.

A program of EDM and MDM measurements for ${\varLambda } ^+_{c}$, $\varXi ^+_{c}$ charm baryons, $\overline{\varXi }^+_{b}$, $\overline{{\varOmega }}^+_{b}$ beauty antibaryons, and $\overline{\varXi }^+$, $\overline{{\varOmega }}^+$ strange antibaryons, has been discussed. The feasibility of the experiment based on the LHCb detector has been assessed relying on both parameterised and Geant4 simulations along with a geometrical model of the detector. Sensitivities for $10^{15}$ $\,\mathrm {PoT}$ could be reached within a few weeks of dedicated detector operations spanned over several years at a flux of $5\times 10^8{p}/{\,\mathrm {s}}$. The possibility of a dedicated experiment a covering larger pseudorapidity region, able to afford higher proton fluxes and longer data taking periods, has also been discussed. For the LHCb layout, optimal bent crystal parameters are determined to be 7$\,\mathrm {cm}$ (5$\,\mathrm {cm}$) length and 14$\,\mathrm {mrad}$ (15$\,\mathrm {mrad}$) bending angle for $\mathrm{Si}$ ($\mathrm{Ge}$), whereas for the dedicated experiment are found to be 12$\,\mathrm {cm}$ (7$\,\mathrm {cm}$) and 7$\,\mathrm {mrad}$ (8$\,\mathrm {mrad}$). In all cases, $\mathrm{Ge}$ crystals provide enhanced sensitivity.

This unique physics program would provide important experimental anchor points for QCD calculations and searches for physics beyond the SM. In the case of charm and beauty baryon EDM the limits would be better than current indirect bounds based on the neutron EDM [11, 13, 16], extending the new physics discovery potential of the LHC.

## Discrete ambiguities

From Eqs. (6), (7) and (8) we observe that, if all the three components of the final polarization vector $\mathbf {s}$ are measured, the *g* and *d* factors can be extracted along with the initial polarization $s_0$, up to discrete ambiguities. If $\{s_0,~g',~d\}$ is a solution, where $g'=(g-2)/2$ is the anomalous magnetic moment, then

31 $$\begin{aligned}&\left\{ -s_0, ~g'\pm \frac{n\pi }{\gamma \theta _C}, ~d\left( 1\pm \frac{n\pi }{\gamma \theta _C}\frac{1}{g'} \frac{\cos \varPhi -1}{\cos \varPhi +1}\right) \right\} ,~\nonumber \\&\left\{ s_0, ~g'\pm \frac{m\pi }{\gamma \theta _C}, ~d\left( 1\pm \frac{m\pi }{\gamma \theta _C}\frac{1}{g'}\right) \right\} ~, \end{aligned}$$

are also solutions, with *n* (*m*) an odd (even) integer. Performing a simultaneous fit in bins of $\gamma$ to the angular distribution described in Eq. (26), will resolve the ambiguity.

## Footnotes

1 The spin-polarization vector is defined such as ${\mathbf {s}} = \langle {\mathbf {S}} \rangle / (J \hbar )$, where ${\mathbf {S}}$ is the spin operator.
